# Supplementary material for: ‘In fact, that’s when I stopped using contraception’: a qualitative study exploring women’s experiences of using contraceptive methods in KwaZulu-Natal, South Africa
Source: BMJ Open. 2023 Apr 20;13(4):e063034. doi: 10.1136/bmjopen-2022-063034 (PMC10124203; doi:10.1136/bmjopen-2022-063034)
Supplement: Author's manuscript [file bmjopen-2022-063034.draft_revisions.pdf]

BMJ Open

BMJ Open is committed to open peer review. As part of this commitment we make the peer review history of every article we publish publicly available.

When an article is published we post the peer reviewers' comments and the authors' responses online. We also post the versions of the paper that were used during peer review. These are the versions that the peer review comments apply to.

The versions of the paper that follow are the versions that were submitted during the peer review process. They are not the versions of record or the final published versions. They should not be cited or distributed as the published version of this manuscript.

BMJ Open is an open access journal and the full, final, typeset and author-corrected version of record of the manuscript is available on our site with no access controls, subscription charges or pay-per-view fees (http://bmjopen.bmj.com).

If you have any questions on BMJ Open's open peer review process please email info.bmjopen@bmj.com

BMJ Open

"In fact, that's when I stopped using contraception": A qualitative study of the experiences of sexually active women regarding contraceptive use in KwaZulu-Natal, South Africa

Journal:	*BMJ Open*
Manuscript ID	bmjopen-2022-063034
Article Type:	Original research
Date Submitted by the Author:	21-Mar-2022
Complete List of Authors:	Hlongwa, Mbuzeleni; University of KwaZulu-Natal College of Health Sciences, Nursing and Public Health<br>Mutambo, Chipo; University of KwaZulu-Natal College of Health Sciences<br>Hlongwana, Khumbulani; University of KwaZulu-Natal College of Health Sciences, Nursing and Public Health; University of KwaZulu-Natal College of Health Sciences, Public Health Medicine
Keywords:	Public health < INFECTIOUS DISEASES, Sexual and gender disorders < PSYCHIATRY, REPRODUCTIVE MEDICINE

SCHOLARONE™
Manuscripts

I, the Submitting Author has the right to grant and does grant on behalf of all authors of the Work (as defined in the below author licence), an exclusive licence and/or a non-exclusive licence for contributions from authors who are: i) UK Crown employees; ii) where BMJ has agreed a CC-BY licence shall apply, and/or iii) in accordance with the terms applicable for US Federal Government officers or employees acting as part of their official duties; on a worldwide, perpetual, irrevocable, royalty-free basis to BMJ Publishing Group Ltd ("BMJ") its licensees and where the relevant Journal is co-owned by BMJ to the co-owners of the Journal, to publish the Work in this journal and any other BMJ products and to exploit all rights, as set out in our licence.

The Submitting Author accepts and understands that any supply made under these terms is made by BMJ to the Submitting Author unless you are acting as an employee on behalf of your employer or a postgraduate student of an affiliated institution which is paying any applicable article publishing charge ("APC") for Open Access articles. Where the Submitting Author wishes to make the Work available on an Open Access basis (and intends to pay the relevant APC), the terms of reuse of such Open Access shall be governed by a Creative Commons licence – details of these licences and which Creative Commons licence will apply to this Work are set out in our licence referred to above.

Other than as permitted in any relevant BMJ Author's Self Archiving Policies, I confirm this Work has not been accepted for publication elsewhere, is not being considered for publication elsewhere and does not duplicate material already published. I confirm all authors consent to publication of this Work and authorise the granting of this licence.

For peer review only

"In fact, that's when I stopped using contraception": A qualitative study of the experiences of sexually active women regarding contraceptive use in KwaZulu-Natal, South Africa

Mbuzeleni Hlongwa[1, 2], Chipo Mutambo[1], Khumbulani Hlongwana[1]

[1]Discipline of Public Health, School of Nursing and Public Health, University of KwaZulu-Natal, Durban, South Africa.

[2]Burden of Disease Research Unit, South African Medical Research Council, Cape Town, South Africa

Corresponding author:
Mbuzeleni Hlongwa
Discipline of Public Health Medicine,
School of Nursing and Public Health, University of KwaZulu-Natal, Howard College Campus, Glenwood, Durban, 4041, South Africa
hlongwa.mbu@gmail.com

For peer review only

Abstract

Objectives: This study explored the experiences of women of reproductive age in the use of contraceptives, in relation to their sexual behaviour in KwaZulu-Natal, South Africa.

Setting: In October 2021, we conducted a qualitative study at Umlazi Township in KwaZulu-Natal province, South Africa, through face-to-face in-depth interviews.

Participants: Fifteen women from four primary health care facilities were recruited through a combination of convenience and criterion-based sampling techniques. Using NVivo version 11, two skilled researchers independently conducted thematic data analysis, as a mechanism for quality assurance, before the results were collated and reconciled.

Results: The study included 15 female participants, aged between 18 and 35 years, of whom two-thirds were aged 18-24 years. We found that women were concerned about unpleasant contraceptive side effects such as prolonged or irregular menstrual periods, bleeding, weight gain, and/or severe pains. Some women stopped using their preferred contraceptive method or opted for a different contraceptive method due to undesirable side effects or contraceptive stock outs. Women also stated that they were not adequately counselled or informed on the use or potential negative effects of various contraceptive methods available at health care facilities.

Conclusions: Interventions aimed at reducing contraceptive stock outs are required to ensure that women are empowered to choose contraception based on their own preference, convenience, and/or experience. It is imperative that counselling on contraceptive methods' side effects be improved, to ensure that women have freedom to make informed decisions about their preferred method, proper management of side effects, and to assist them with method switching as needed, instead of discontinuation.

Keywords: Women, experiences, contraceptive use, sexual behaviour, KwaZulu-Natal, South Africa

For peer review only

Introduction

Contraception is one of the most important public health interventions which respond to sexual and reproductive health needs of women, thereby enabling them to plan their pregnancies and decide on the number of children they desire (1). The consistent and correct use of contraception has far-reaching benefits to both individuals and societies, including the reduction of pregnancy-related morbidity and mortality, termination of pregnancies (ToPs), improving educational opportunities, and empowerment of women (2, 3). Furthermore, contraceptive use positively affects the overall health of women of reproductive health, as it empowers them to autonomously make decisions regarding their own sexual and reproductive health (4, 5). Studies have shown that empowered and educated women are more likely to make informed decisions about the use of contraception (6, 7).

Despite the notable improvements in the uptake of contraceptives by women of reproductive age globally, the Sub-Saharan African (SSA) region continues to record a high proportion of women who experience unplanned pregnancies every year (8). In SSA, unmet contraception needs for women of reproductive age continue to be a public health concern, with a substantial number of these women being unable to access and use their preferred methods of contraception, which jeopardizes their desire to circumvent unplanned pregnancies (9). In the context of this study, an unmet need for contraception is defined as an inconsistent or incorrect use of a contraceptive method by a sexually active woman of reproductive age, for preventing unwanted pregnancy (10, 11). The rate of unmet needs for contraceptive use is more than 20% among sexually active women in SSA (12, 13).

The 2016 Demographic and Health Survey report showed that 55% and 60% of married women and sexually active women, respectively, used contraceptives in South Africa (10). At least 15% of women who are in-union, have an unmet need for contraception to prevent unplanned pregnancy in the country, and the figure is slightly higher (19%) for sexually active women; meanwhile, KwaZulu-Natal (KZN) accounts for 18% and 21% of the national figures (unmet need for contraception), respectively, among in-union and sexually active women (10). In South Africa, more than 100 000 terminations of pregnancy were reported in designated facilities in the 2016/2017 financial year, with KZN accounting for 15% of these (14). Most ToPs are conducted illegally, contributing to high maternal morbidity and mortality rates (3,

For peer review only

15, 16). High rates of unplanned pregnancy have also been reported among women diagnosed with HIV in South Africa (17). Risky sexual behaviours are also common among young women in this country, as well as in KZN, including early sexual debut (below 15 years), sex under the influence of alcohol or drugs, multiple sexual partners, and inconsistent condom use, all of which contribute to high levels of unplanned pregnancy and sexually transmitted diseases, including HIV (18-23).

Factors contributing to high rates of unplanned pregnancy in limited resource settings, including South Africa, are well documented. Insufficient knowledge of contraception, gender inequality, intimate partner violence, poverty, and inconsistent and incorrect use of contraception, are some of the factors (8, 24, 25). Low levels of contraceptive use have been associated with age, low education level and low socioeconomic status, limited knowledge and inaccessibility of contraceptive methods, and resource-limited rural residential settings (20, 26-28). Health system challenges, including contraception stock-outs, long waiting times, and negative attitudes displayed by some health care providers, have also been reported to deter women, especially young women, from accessing contraceptive services from health care facilities, thereby contributing to low or inconsistent uptake of contraceptives (29, 30). Studies have shown that high rates of contraceptive users discontinue using contraception within a year of starting to use one of the methods, while others switch to a different method due to side effects, as well as stock-outs of preferred methods in local public health care facilities (10, 31, 32).

Despite these challenges, the South African government has demonstrated commitment to ensuring universal access to contraception by women of reproductive age. In South Africa, only 46% of births occurring within a five-year period preceding the SADHS 2016, were wanted at the conception time, while 34% and 20%, respectively, were mistimed and unwanted (10). The use of qualitative methods to explore women's experiences of contraceptives will deepen our understanding of this phenomenon; this will in turn guide the development and/or the strengthening of interventions aimed at improving contraceptive use among women of reproductive health in KwaZulu-Natal, and other comparable resource-limited settings.

For peer review only

Methods

Study setting

Umlazi, a township populated with more than half a million people, is located in the province of KZN (33) and is part of the EThekwini Metro, which has the largest number of people on lifelong anteretroviral therapy (ART) in the province (34). Umlazi has 10 public health care clinics and one public health hospital. Four public health care clinics, spread across various parts of Umlazi, participated in the study.

Study design

We explored women's experiences of contraceptives in relation to their sexual behaviour, using an exploratory descriptive qualitative study design (35). We generated data in October 2021, through face-to-face in-depth interviews with women of reproductive age accessing health care services, from four facilities in Umlazi.

Sampling

We employed a combination of convenience and criterion-based sampling strategies, to identify potentially eligible women from four primary heath care facilities in Umlazi Township, KZN. We used convenience sampling in the sense that we had no prior knowledge of the information richness of our participants, but conveniently recruited from those women who presented themselves to the participating health facilities, during the period of data collection. After conveniently identifying the potentially eligible women, we drew from criterion-based sampling techniques, by screening whether woment were or not: (a) of reproductive age (18–49 years); (b) residing in Umlazi Township; (c) sexually active (women who had sexual intercourse within three months preceding data collection); and (d) using contraception or had used contraception within three months preceding data collection. Women who were outside the age brackets (18-49 years), pregnant, or sexually inactive, were excluded from the study. Interviews were conducted after services had been rendered by the facility.

Data collection tool

We developed an in-depth interview guide in the English language and then translated it into IsiZulu, which is the dominant language used in Umlazi. A female research assistant with a track record of conducting interviews and qualitative research, generally, was recruited and provided with refresher training prior to collecting data. The research assistant was fluent in

For peer review only

both English and IsiZulu languages. Interviews were audio-recorded (with participants' permission). To minimize any inconsistencies during the data collection period, the interview guide was pre-tested with five participants who did not form part of the study.

Data collection

Data was collected iteratively to ensure that researchers engaged with preliminary data analysis of the information collected, learn emerging themes, identify gaps in the data, and adapt the data collection process for subsequent interviews. Each interview lasted for 30 to 60 minutes. Data saturation was reached at 15 interviews. At least five potential participants refused to participate in the study when they were approached. The lead author held regular debriefing meetings with the research assistant to discuss field experiences, challenges, emergent issues, lessons learned, and how she affected and/or was affected by the interviews. The scope of inquiry of the interviews focused on four key components of contraception, namely: (i) awareness about different contraceptive methods, (ii) access (availability/stock-outs of preferred contraceptive methods, counselling), (iii) uptake (key considerations in deciding whether or not to start using contraception), and (iv) adherence/continuation (key considerations in deciding whether or not to continue or discontinue using contraception, including side effects, violence, stigma, discrimination, judgements).

Data analysis

Using NVivo version 11, two researchers independently conducted the analysis from data coding to the development of themes, and this was done iteratively, guided by Richie and Spencer's framework (36). The framework outlines the following stages for conducting qualitative data analysis: (a) familiarisation with the data through reading all the transcripts and listening to the audio recordings; (b) generating initial codes using an open coding method, where each segment of data relevant to this study's research objective was coded; (c) development of a thematic framework extracting key themes from the coded data; (d) application of the thematic framework to all the data; (e) charting of the data, enabling systematic comparisons between data sets; and (f) analysis of the charts for patterns and associations between and within each unit of analysis.

For peer review only

Data quality

To ensure rigor and accuracy, comparative data analysis was conducted by two skilled researchers, who independently read all the transcripts to gain an understanding of the content and scope of data collected, prior to data coding. The outcome of coding was verified, cross-checked, and thoroughly discussed between the two members, to ensure that the research question was answered. We used the COREQ checklist to ensure that the study adhered to quality standards for reporting qualitative study findings (37).

Ethical considerations

Ethical approval and gatekeeper permission were obtained from the University of KwaZulu-Natal (UKZN) Biomedical Research Ethical Committee (BREC) (Ref No: BE424/18) and the Department of Health's National Health Research Database (NHRD) (Ref No: KZ_2018009_013), respectively. The EThekwini District's Ethical Review Committee also approved the study. The study was also supported by the participating health facilities. All participants who volunteered to participate in the study signed an informed consent form prior to their participation. The privacy and confidentiality of participants were protected.

Patient and Public Involvement

Patients or the public were not involved in the design, or conduct, or reporting, or dissemination plans of our research

Results

Background characteristics of study participants

The study included 15 women, aged between 18 and 35 years, with a median age of 23 years (Table 1). All participants were Black African women, with more than two-thirds (n=10) aged 18-24 years. The majority (n=12) had attained a secondary level of education and nearly half (n=7) were unemployed. Almost all participants (n=14) were not married.

For peer review only

Table 1: Demographic characteristics of participants, Umlazi Township, KwaZulu-Natal, South Africa, 2021

Characteristic	Participants n=15
Age (years)	18-49
Median (IQR)	23 (19-26)
Age categories, n (%)	
18-24 years	10 (67)
25-49 years	5 (33)
Population group, n (%)	
Black African	15 (100)
Level of education, n (%)	
Secondary	12 (80)
Tertiary	3 (20)
Employment status, n (%)	
Unemployed	7 (47)
Employed	4 (27)
Studying	4 (27)
Marital status, n (%)	
Not married	14 (93)
Married	1 (7)

Key themes

The following key themes emerged from the analysis: early sexual debut and unplanned pregnancy; concerns over contraceptives efficacy and side effects; contraceptive methods' stock-outs, inconsistent or incorrect use of contraceptive methods; inadequate counselling on contraceptive methods; misconceptions about contraception; and risky sexual behaviours.

Early sexual debut and fear of unplanned pregnancy

Most participants reported that they started to use contraceptive methods at a young age, and they attributed their decisions to an early sexual debut. Some participants' use of contraceptives dated back to their schooling days and the main motivator was pregnancy avoidance.

"For me to decide using contraception, I was seeing that I was still young and I was still attending school. I was also thinking for my mom that she's working this side and having a baby while studying was not right. So I decided that since I'm having sex, I should decide that instead I should then use contraception". (24-year-old, unemployed, with secondary-level education)

"I was 17 years at the time and I started using contraception. I was starting to have sex then I used contraception". (19-year-old, unemployed, with tertiary-level education)

For peer review only

"No, the first time I had sex, I was still young. I was 15 years old". (29-year-old, employed, with secondary-level education)

Due to an early sexual debut, some women reported being surprised by their first unplanned pregnancy, which occurred while they were very young. Becoming pregnant after the first time having sexual intercourse was particularly surprising for them, as they thought pregnancy occurs after several sexual encounters.

"Even with this first birth, it was a big mistake. I did not plan to have a child. It was my first time having sex. I didn't even know I was pregnant. My mother is the one who noticed…. I couldn't believe it…. It was my first time having sex, I had not planned to get pregnant. Even my boyfriend had not planned it. He was also surprised". (18-year-old studying at secondary-level education)

I didn't plan to get pregnant. That mistake happened when I did not use a protection. And then I got a child. I started having sex when I was doing grade 12". (20-year-old, unemployed, with secondary-level education)

One woman indicated that she experienced unplanned pregnancy while she was actively using a contraceptive method, and the health care providers responded by saying that the injectable contraceptive method is not 100% effective.

"Let me see, 2018 I did use the injection. I used the injection throughout 2018. I was active on injection because I didn't want another child. And then I got pregnant while I was using the injection. And the nurse said that the injection is not 100% effective… I asked them, how, it's not 100% then why are we using it? The nurse just said that the injection is not 100% protective. I was using contraception when I got pregnant. I didn't plan to have a child". (23-year-old, unemployed, with secondary-level education)

Concerns over contraceptives' efficacy and their side effects

Concerns over the effectiveness of using contraceptive methods were raised, with some women reporting that they do not trust that the contraceptives are effective enough to prevent someone from getting an unplanned pregnancy.

For peer review only - http://bmjopen.bmj.com/site/about/guidelines.xhtml

"Yoh ehm, I think that because there's many different contraceptive methods, what I know about the injection is that it reduces chances of getting pregnant. But it is not 100% accurate…. I will continue using a condom because I know that the injection is not 100% sure." (18-year-old studying at secondary-level education)

"Even pills, I'm scared of them. I got pregnant while I had used the morning after pills. I don't trust the pills". (26-year-old, unemployed, with secondary-level education)

In addition to contraceptive mistrust, there were reports of unpleasant contraceptive side effects from some of the participants. The most common side effect reported by the participants, was prolonged menstrual periods resulting from using injectable contraceptives.

"The injection made me have prolonged periods than usual. I usual go on periods for three days, but after taking the injection my periods would last for seven days… I was tired of being on periods all the time. My periods lasted longer than usual. I'd keep having periods. Then it would stop eventually, but then again the following months when it's time for my periods, they would last longer… The next month, same thing". (19-year-old, unemployed, with tertiary-level education*)*

"I went on a prolonged menstrual cycle, my periods started from the 2nd to the 12th August… I don't know what is going on, I don't know…But the injection didn't treat me well. I had started with the two months injection. But I stopped using the two months injection because it would make me go on periods at any time. It made me bleed at any time". (19-year-old studying at secondary-level education)

Some women indicated that using the injectable contraceptives made them bleed heavily, or that the injectable interfered with their menstrual cycle, and resulted in inconsistent menstrual periods.

She [nurse] had given me the 3 months injection. The three months injection was making me bleed a lot. My bleeding couldn't stop. Then I did some research and asked from others and they said that it happens that the injection would not respond well on your body". (20-year-old, unemployed, with secondary-level education)

"It was not treating me well. It would make me go on periods at least twice in one month. I would go on periods for three or four days but twice a month. In the beginning it treated me well. Then it changed later on". (25-year-old, employed, with secondary-level education)

For peer review only

Women reported that using contraception contributed to changes in their appetite for food, thereby affecting their weight. Some experienced weight gain and others lost weight.

"I would eat a lot. I would eat every now and then. And if I wanted food, I really wanted food, like someone who's pregnant". (20-year-old, unemployed, with secondary-level education)

"…when I started with the contraceptives, I gained a lot of weight and I was oily, like my face was messed up so I stopped and then after a year I went back [re-started contraception]…". (22-year-old, employed, with secondary-level education)

"I started by using the three months injection, but it made me lose weight so bad, then I switched from it to the two months injection". (25-year-old, employed, with secondary-level education)

Some women indicated that they experienced severe pains, as a result of using the injectable contraceptive method.

"No, but I've used injection before and the injection didn't treat me well. I always had problems in my bladder and I decided to stop using it. I would have unusual pains, which is something I had never experienced before I started using contraception". (27-year-old, unemployed, with secondary-level education)

Participants' reactions to side effects

Some participants reacted to side effects by simply discontinuing contraception.

"The injection made me have [more] extended periods than usual… So I didn't even ask, I just decided to stop using contraception. In fact, that's when I stopped using contraception. I stopped using contraception last year or last-of-last year". (19-year-old, unemployed, with tertiary-level education)

"At the clinic they told me there's this kind of injection and I should try it. But I then stopped it when I experienced these side effects. I would also get sick. I was supposed to go on periods. But because of the injection I wouldn't go on periods. I stopped it". (27-year-old, unemployed, with secondary-level education)

Others reacted to side effects by simply switching to a different contraceptive method.

For peer review only

"Then I came back and told the nurse about the side effects I was experiencing while using that injection. I then asked her to switch me to a different method. And then she switched me to a two-months injection". (20-year-old, unemployed, with secondary-level education)

"I told them that I'd like to switch from using this contraception because I had started using the pills for a short while. So I knew how they worked. Then I asked them to change me to the pills. Now I only go on periods once a month". (35-year-old, unemployed, with tertiary-level education)

Stock-outs of preferred contraceptive methods and participants' reactions

Participants reported stock-outs of their preferred contraceptive methods as a pervasive challenge in the health care facilities.

"Yes, even last week they said they were out of stock, especially the three months injection, they only had the two month's one". (18-year-old studying at secondary-level education)

"…sometimes you come here and find that injection is not available… I don't know what can they do to improve the situation. I sometimes ask nurses why they don't have the injections and they say that it is the department that is not delivering them". (20-year-old, unemployed, with secondary-level education)

One participant saw Implanon as a potential solution to the challenge of stock-outs, as this method does not require her to make regular visits to the facility for contraceptive purposes.

"…so, I'm considering using it [Implanon] because the injection is sometimes not available at the clinic. And they said at the clinic that if the injections are finished again, they don't know when it will be available again…". (23-year-old, unemployed, with secondary-level education)

"When I got to this clinic, I was told that injectable contraceptive methods is no longer available. They told me that there is the implant contraceptive method. But I'm scared of implant. I ended up stopping using contraception because I was scared of using the implant. I'm still going to think about the implant but it is scary…I came [to the clinic] on September 15, and I asked for implant and they said that I should come back another day….They do tell you that it's not that they don't offer it, it's just out of stock, you should come back". (24-year-old, unemployed, with secondary-level education)

For peer review only

Changing to an alternative contraception method was one of the options pursued by some participants whenever they were unable to receive their preferred contraceptive method, while others opted to wait for their preferred contraception. Those who opted to wait for their contraception of choice, had to modify their lifestyle during the period of being not protected by contraception.

"I continued using the three months injection. But it would happen that sometimes they would not have it at the clinic. Then I would be switched to the two months injection". (20-year-old, unemployed, with secondary-level education)

"I decided to stop. I didn't take them. I stopped using contraceptives. My date was on the 12th September. I came here and I was told that the injection is not available. Then, again I came back on the 18th September. And they told me again that the injection is not available. Then I waited. Then I received a message from my friend who told me that she was at the clinic and that the injection is now available. I think it was last week. Then I came back and took the injection". (20-year-old, unemployed, with secondary-level education)

"For me I don't want to use something else when I'm still going to use the implant. I don't want too many in my body. At least if I use one. I should not mix. I should just be patient for the implant. I should be able to control myself. For now, I'm trying to avoid having sex, since August". (24-year-old unemployed, with secondary-level education)

The contraception stock-out was reported to have far-reaching implications on women, as it does not only make them vulnerable to unplanned pregnancies, but also causes emotional discomfort.

"During that time the injection is not available, you are not comfortable. You are scared. It is hard when the injection is not available". (20-year-old, unemployed, with secondary-level education)

Others were discouraged to continue using contraception due to stock-outs.

"You'll end up getting discouraged if you don't get the contraceptive methods you need. All they need to do is to ensure that the preferred contraceptive methods are always available". (35-year-old, employed, with tertiary-level education)

For peer review only

Inconsistent or incorrect use of contraceptive methods

Participants indicated that they sometimes could not consistently use contraceptives, as some of them missed their re-injection appointments. This resulted in them experiencing an unplanned pregnancy.

"…When my re-injection date had passed, as I was taking a break, so that my blood will flow and the side effects would stop, I then got pregnant. I had planned to wait for a month. Then I would start the following month. It was stupid I don't know how [laughs]. And then during that period I became pregnant. It just happened so quickly. I couldn't believe it. I couldn't believe that I was really pregnant". (23-year-old, unemployed, with secondary-level education)

"I do have a child. I didn't stop using contraception, but I got the child after I skipped my date for re-injection. It just happened that I got pregnant". (25-year-old, employed, with secondary-level education)

Most participants showed less preference for the contraceptive pills, fearing that they would not be able to maintain or use them consistently or correctly.

"…what made me stop using them [contraceptive pills] is because I used to miss them because you have to take them at the same time daily, I stopped taking them… I stopped using them because I kept missing the time whereas you are supposed to take them at the same time". (19-year-old studying at secondary-level education)

"I've never used something else. I've only used the injection. Because I cannot use the pills because I would forget them. I forget the pills I cannot use them". (23-year-old, unemployed, with secondary-level education)

Some participants reported instances where health care providers either inserted the implant incorrectly, or inserted it into a pregnant woman, as they hardly ever checked a woman's pregnancy status before inserting the implant.

"…and I also got pregnant while the implant was inserted in me… Maybe they inserted it while I was already pregnant. They didn't check my pregnancy status. They didn't run pregnancy tests. They just inserted it". (19-year-old studying at secondary-level education)

For peer review only

"I had pregnancy symptoms. I would vomit. I would experience things that I didn't understand. Then I went back to the clinic and I was told that the implant was not inserted correctly. Then they started telling me about the possible risks since I am already pregnant. That I might get a miscarriage. And I did get a miscarriage. I don't know if that's what caused the miscarriage. After they removed the implant, I had already lost hope because I was bleeding". (19-year-old studying at secondary-level education)

Lack of counselling on contraceptive methods

Participants indicated that they were not counselled or given information regarding the use of, and the possible side effects, of various contraceptive methods offered at health care facilities.

"…the nurse did not explain anything to me. She did not tell me how it works. I do have some questions about using contraception. I want to ask that since they are different, there's one for three months and another one for two months – what's the difference between them? That's all I want to ask". (18-year-old studying at secondary-level education)

"She didn't even ask me which method I wanted to use or what. She didn't and I couldn't ask questions. I didn't even know which injection she was giving to me [after giving birth at the hospital]. She didn't say anything. She just calls your name and then gives you the injection….. It got me confused. Because I wanted to use a different method. But I only realized what they had given me when I was reading the card because it was written there. And there was nothing I could still do". (20-year-old, unemployed, with secondary-level education)

"…there was no opportunity because in the room we were getting injections from, we were many so there was no space for discussing or asking questions. So you'd get the injection and go home. You didn't have time to discuss things or ask questions". (24-year-old, unemployed, with secondary-level education)

Misconceptions about contraception

Some participants raised concerns about the possibility of contraception impairing future fertility, with others preferring to wait until they got their first child before using contraceptive method.

"…but the very first time I heard about contraception, I was told by my mother who asked me to use contraception, so that I won't get pregnant. I hadn't had a child at that time. But I refused because I heard people saying that if you use contraception when you haven't had a

For peer review only

child, it happens that you might not be able to conceive. So I didn't use contraception. I only started using contraception after getting a baby. I have one child". (20-year-old, unemployed, with secondary-level education)

"I only used it for a short period because I was told that it's dangerous to use the injection when you don't have a child because it completely prevents you from getting pregnant in future….". (25-year-old, employed, with secondary-level education)

"I do have questions. I have many questions. Because even now I am concerned. Some people do say that the injection makes you infertile even after you have stopped using it. You won't go on periods. Now that it's been months without seeing my periods, I'm scared". (19-year-old, unemployed, with secondary-level education)

Spontaneity of sexual activities

Some women reported that they engaged in spontaneity of sex, whereby they find themselves engaging in unprotected sex without any anticipation or preparation, some of which resulted in sexually transmitted infections.

"That's where the mistake was [laughs]. We didn't use a condom. When I was visiting him, I hadn't planned to have sex with him. It just happened. I just gave him one round". (18-year-old studying at secondary-level education)

"I just went to check for HIV. I already knew that I was running (promiscuous). I didn't get sick or anything. And they told me that I am HIV positive… things just got too fast. I was even shocked that how did it happen that I would have sex with someone so quick. It was like there was something that said that I want to teach you a lesson. Condom is really important". (26-year-old, unemployed, with secondary-level education)

"That has happened… Sometimes we run out of condoms. Sometimes it's there and it just happens that we don't use it… I came to the clinic and I was told that I have drop…My partner also came to check and found that he also had it…". (23-year-old, unemployed, with secondary-level education)

Discussion

Understanding the disparities in contraceptive use in South Africa, requires a thorough investigation of factors contributing to contraceptive uptake and discontinuation. In this

country, the use of contraception is often limited to a few methods, such as the injectable, oral contraceptive pills, and condoms (10). Availability of contraceptive methods at primary health care facilities varies, with injectable, oral contraceptive pills, and condoms being the most common, despite the stock-outs and side effects being regularly reported.

In this study, we adopted a qualitative approach to exploring women's experiences of contraceptive use, in relation to their sexual behaviour in KwaZulu-Natal, South Africa. Our study participants shared crucial insights for understanding the challenges women face, when accessing and using contraceptive methods in Umlazi Township, KZN. Our results show that concerns over contraceptives' efficacy, contraceptive method side effects, contraceptive methods' stock-outs, and lack of counselling on contraceptive methods, affect the uptake of contraceptives in the study setting, and this may have some relevance for other comparable settings.

Side effects play an important role in a woman's decision as to whether to continue or discontinue using contraception, as has been reported in other studies (38, 39). Similar to our study, prolonged or irregular menstrual cycle patterns have also been reported as one of the most common side effects that influence women to discontinue contraceptive use (39-41). Both the injectable users and implant users have concerns about bleeding side effects, which are known to have contributed to discontinuation of contraception in similar settings (39). Contraceptive discontinuation has dire implications, including unplanned pregnancy. Therefore, women should be empowered to avoid unplanned pregnancies, by identifying alternative contraceptive methods that meet their individual needs.

There has been concern about the growing number of women who return to clinics for implant removal, only after a few months of insertion, due to changes in bleeding patterns or excessive bleeding (40, 42, 43). Side effects, notably prolonged or irregular bleeding, have also been reported by health care providers as the most common reason for early Implanon discontinuation (40). In addition to this, other factors have been reported to influence women to remove the implant, including incorrect positioning and low quality of care (44-46). Incorrect positioning of the implant was also reported in this study as an important factor for its discontinuation. The implant protects against pregnancy for up to three years before it needs

For peer review only

to be replaced, and this reduces frequent clinic visits among users (47). The growing number of women removing the implant given the side effects, suggests the need for pre-insertion counselling, proper management, and empowerment of women through information regarding alternative contraceptive options for better decision-making (43).

Contraceptive stock-outs occur when one or more contraceptive methods are not available at a health care facility that routinely provides that method. Contraception stock-outs are one of the main factors contributing to contraceptive switching and discontinuation among women (48, 49). Contraceptive methods should be available at all levels of health care, but not all health care facilities offer the full mix of modern contraceptive methods. Women have different contraceptive method preferences and experiences, therefore, a wide variety of contraceptive methods should always be available; this ensures that women are empowered to choose contraception based on their own preference, convenience, and/or experience. Given the high rates of contraceptive method stock-outs in South Africa, unemployed women are likely to suffer the most, due to financial constraints, whereas women with financial resources, may be able to access preferred contraceptive methods through the private sector.

Health care providers play an important role in ensuring access to contraceptive methods among women. As such, health care providers have an important responsibility in educating women and providing complete information about possible side effects, and the effectiveness of their preferred method. It is imperative that counselling on side effects be improved, to ensure that women have freedom to make informed decisions about their preferred method, and to assist them with method switching as needed. However, counselling when receiving family planning services, is limited in South Africa (32).

In this study, some women had concerns about contraception impairing future fertility. There were also concerns among some women over the effectiveness of contraceptive methods, due to experiencing pregnancy while actively using contraception. These concerns may be addressed through improved counselling and support from health care providers. Some women were concerned about inconsistent or incorrect use of contraceptive methods. These results

For peer review only

suggest the need to improve counselling to promote contraception awareness among women, and to clarify any myths and complexities.

Much more effort is needed for counselling, to ensure that women are aware of potential adverse effects from contraceptive use, so as to allow them to make informed decisions. However, a single face-to-face counselling appointment in a busy facility may not be enough to convey all of the information a woman requires about: the reproductive cycle, returning to fertility after discontinuing a method, potential drug–drug interactions, the need for dual protection, and so on (32). In addition to traditional face-to-face interactions between health care providers and contraceptive users, innovative approaches, such as internet-based sources of information, text message reminders, and brochures are needed to improve women's comprehension of how contraceptive methods work (32, 50, 51).

Our study has important limitations to note. The findings of this study were sought from participants' self-reported sexual and reproductive health information, rendering them prone to social desirability bias. Lastly, due to the qualitative nature of the study, the fact that this study was confined to limited health care facilities and participants, meant that our study findings cannot be generalised to other settings. However, the study provides important insights regarding the perceptions and experiences of contraceptive use among sexually active women in Umlazi Township, KwaZulu-Natal, South Africa.

Conclusion

This study contributes to our understanding of women's concerns and challenges arising from accessing and using contraception in Umlazi Township, KwaZulu-Natal. Our findings illustrate that concerted efforts are urgently required to address women's concerns regarding the side effects arising from using contraceptive methods, as well as contraceptive stock-outs, given the dire implications these may have on contraceptive discontinuation and subsequent unplanned pregnancy. The provision of comprehensive counselling services to support women who are having short-term side effects is paramount, to ensure that they are able to deal with side effects, or switch to a different method instead of completely discontinuing contraceptive use, to avoid unplanned pregnancy.

For peer review only

Declarations

Ethics approval and consent to participate: Ethics approval was obtained through the Biomedical Research Ethics Committee (BREC) from the University of KwaZulu-Natal (Ref No: BE424/18). Approval was obtained through the National Health Research Database (NHRD) from the KwaZulu-Natal Provincial Department of Health (Ref No: KZ_2018009_013).

Consent for publication: Not applicable.

Availability of data and materials: All the data analysed and reported in this paper will be made available upon request.

Competing interest: The authors declare that they have no competing interests.

Funding: The work reported herein was made possible through funding by the South African Medical Research Council through its Division of Research Capacity Development under the **BONGANI MAYOSI NATIONAL HEALTH SCHOLARS PROGRAM** from funding received from the South African National Treasury. The content hereof is the sole responsibility of the authors and does not necessarily represent the official views of the SAMRC or the funders.

Authors' contributions: MH conceptualized and designed the study, as well as prepared the initial draft. KH and CM reviewed the manuscript. All the authors approved the final version of the manuscript.

Acknowledgements: The authors would like to thank the School of Nursing and Public Health, University of KwaZulu-Natal, Durban, South Africa and the Division of Research Capacity Development at SA MRC.

For peer review only

References

1. Bongaarts J, Cleland JC, Townsend J, Bertrand JT, Gupta MD. Family planning programs for the 21st century: rationale and design. 2012.
2. Michalow J, Chola L, McGee S, Tugendhaft A, Pattinson R, Kerber K, et al. Triple return on investment: the cost and impact of 13 interventions that could prevent stillbirths and save the lives of mothers and babies in South Africa. . BMC Pregnancy and Childbirth 2015;15(1):1-13.
3. Darroch JE, Woog V, Bankole A, Ashford LS. Adding it up: costs and benefits of meeting the contraceptive needs of adolescents. 2016.
4. Ganatra B, Faundes A. Role of birth spacing, family planning services, safe abortion services and post-abortion care in reducing maternal mortality. Best Practice & Research Clinical Obstetrics & Gynaecology. 2016;36:145-55.
5. Hameed W, Azmat SK, Ali M, Sheikh MI, Abbas G, Temmerman M, et al. Women's empowerment and contraceptive use: the role of independent versus couples' decision-making, from a lower middle income country perspective. PloS one. 2014;9(8):e104633.
6. Corroon M, Speizer IS, Fotso J-C, Akiode A, Saad A, Calhoun L, et al. The role of gender empowerment on reproductive health outcomes in urban Nigeria. Maternal and child health journal. 2014;18(1):307-15.
7. Tekelab T, Melka AS, Wirtu D. Predictors of modern contraceptive methods use among married women of reproductive age groups in Western Ethiopia: a community based cross-sectional study. BMC women's health. 2015;15(1):1-8.
8. Cleland J, Harbison S, Shah IH. Unmet need for contraception: issues and challenges. Studies in family planning. 2014;45(2):105-22.
9. United Nations. Family Planning and the 2030 Agenda for Sustainable Development. . New York: United Nations, Department of Economic and Social Affairs, Population Division.; 2019.
10. NDoH, SA S, SAMRC, ICF. South Africa Demographic and Health Survey 2016. Pretoria, South Africa, and Rockville, Maryland, USA: : National Department of Health (NDoH), Statistics South Africa (Stats SA), South African Medical Research Council (SAMRC), ICF. ; 2019.
11. Sedgh G, Ashford LS, Hussain R. Unmet need for contraception in developing countries: examining women's reasons for not using a method. 2016.
12. Darroch JE, Sully E, Biddlecom A. Adding it up: investing in contraception and maternal and newborn health, 2017—supplementary tables. New York, NY: The Guttmacher Institute. 2017.
13. World Health Organization. Contraception Geneva, Switzerland: World Health Organization; 2019.
14. Department of Health. Pregancy terminations in designated South African facilities. Pretoria: SA National Department of Health; 2017.
15. Hodes R. The culture of illegal abortion in South Africa. Journal of Southern African Studies. 2016;42(1):79-93.
16. Seidu A-A, Ahinkorah BO, Agbemavi W, Amu H, Bonsu F. Reproductive health decision-making capacity and pregnancy termination among Ghanaian women: Analysis of the 2014 Ghana demographic and health survey. Journal of Public Health. 2021;29(1):85-94.
17. Adeniyi OV, Ajayi AI, Moyaki MG, Ter Goon D, Avramovic G, Lambert J. High rate of unplanned pregnancy in the context of integrated family planning and HIV care services in South Africa. BMC health services research. 2018;18(1):1-8.
18. Hlongwa M, Peltzer K, Hlongwana K. Risky sexual behaviours among women of reproductive age in a high HIV burdened township in KwaZulu-Natal, South Africa. BMC Infectious Diseases. 2020;20(1):1-9.
19. Toska E, Cluver LD, Boyes M, Pantelic M, Kuo C. From 'sugar daddies' to 'sugar babies': exploring a pathway among age-disparate sexual relationships, condom use and adolescent pregnancy in South Africa. Sexual health. 2015;12(1):59-66.

For peer review only

20. Christofides NJ, Jewkes RK, Dunkle KL, Nduna M, Shai NJ, Sterk C. Early adolescent pregnancy increases risk of incident HIV infection in the Eastern Cape, South Africa: a longitudinal study. Journal of the International AIDS Society. 2014;17(1):18585.
21. Atuyambe LM, Kibira SP, Bukenya J, Muhumuza C, Apolot RR, Mulogo E. Understanding sexual and reproductive health needs of adolescents: evidence from a formative evaluation in Wakiso district, Uganda. Reproductive health. 2015;12(1):1-10.
22. Solanke BL. Factors influencing contraceptive use and non-use among women of advanced reproductive age in Nigeria. Journal of Health, Population and Nutrition. 2017;36(1):1-14.
23. Blackstone SR, Iwelunmor J. Determinants of contraceptive use among Nigerian couples: evidence from the 2013 Demographic and Health Survey. Contraception and reproductive medicine. 2017;2(1):1-8.
24. Maharaj P. Stalling contraception? Perspectives and experiences of sexually active women and men. Agenda. 2012;26(2):100-10.
25. Jewkes R, Morrell R, Christofides N. Empowering teenagers to prevent pregnancy: lessons from South Africa. Culture, health & sexuality. 2009;11(7):675-88.
26. Makola L, Mlangeni L, Mabaso M, Chibi B, Sokhela Z, Silimfe Z, et al. Predictors of contraceptive use among adolescent girls and young women (AGYW) aged 15 to 24 years in South Africa: results from the 2012 national population-based household survey. BMC women's health. 2019;19(1):1-7.
27. Digitale J, Psaki S, Soler-Hampejsek E, Mensch BS. Correlates of contraceptive use and health facility choice among young women in Malawi. The ANNALS of the American Academy of Political and Social Science. 2017;669(1):93-124.
28. Nyarko SH. Prevalence and correlates of contraceptive use among female adolescents in Ghana. BMC women's health. 2015;15(1):1-6.
29. Hlongwa M, Tlou B, Hlongwana K. Healthcare providers' knowledge and perceptions regarding the use of modern contraceptives among adolescent girls in Umlazi Township, KwaZulu-Natal province, South Africa. The Pan African Medical Journal. 2021;38.
30. Smith P, Marcus R, Bennie T, Nkala B, Nchabeleng M, Latka M, et al. What do South African adolescents want in a sexual health service? Evidence from the South African Studies on HIV in Adolescents (SASHA) project. South African Medical Journal. 2018;108(8).
31. Blanc AK, Curtis SL, Croft TN. Monitoring contraceptive continuation: links to fertility outcomes and quality of care. Studies in family planning. 2002;33(2):127-40.
32. Lince-Deroche N, Pleaner M, Morroni C, Mullick S, Firnhaber C, Harries J, et al. Achieving universal access to sexual and reproductive health services: the potential and pitfalls for contraceptive services in South Africa. South African Health Review. 2016;2016(1):95-108.
33. Africa SS. Census 2011. Pretoria: Statistics South Africa; 2012.
34. Simbayi L, Zuma K, Zungu N, Moyo , Marinda E, Jooste S, et al. South African National HIV Prevalence, Incidence, Behaviour and Communication Survey, 2017. Cape Town: HSRC; 2019.
35. Parker I. Discourse analysis: Dimensions of critique in psychology. Qualitative Research in Psychology. 2013;10(3):223-39.
36. Ritchie J, Spencer L. Qualitative data analysis for applied policy research. Analyzing qualitative data: Routledge; 2002. p. 187-208.
37. Tong A, Sainsbury P, Craig J. Consolidated criteria for reporting qualitative research (COREQ): a 32-item checklist for interviews and focus groups. International journal for quality in health care. 2007;19(6):349-57.
38. Castle S, Askew I. Contraceptive discontinuation: Reasons, challenges and solutions: Population Council. FP2020. 2015.
39. Keogh SC, Otupiri E, Castillo PW, Chiu DW, Polis CB, Nakua EK, et al. Hormonal contraceptive use in Ghana: the role of method attributes and side effects in method choice and continuation. Contraception. 2021.

For peer review only

40. Adeagbo O, Mullick S, Pillay D, Chersich M, Morroni C, Naidoo N, et al. Uptake and early removals of Implanon NXT in South Africa: Perceptions and attitudes of healthcare workers. South African Medical Journal. 2017;107(10).
41. Veisi F, Zangeneh M. Comparison of two different injectable contraceptive methods: Depo-medroxy progesterone acetate (DMPA) and cyclofem. Journal of family & reproductive health. 2013;7(3):109.
42. Burusie A. Reasons for premature removal of Implanon among users in Arsi zone, Oromia region, Ethiopia, 2013. Reproductive System & Sexual Disorders: Current Research. 2015;4(1):1-6.
43. Mrwebi KP, Ter Goon D, Owolabi EO, Adeniyi OV, Seekoe E, Ajayi AI. Reasons for discontinuation of Implanon among users in Buffalo City Metropolitan Municipality, South Africa: a cross-sectional study. African journal of reproductive health. 2018;22(1):113-9.
44. Zerihun H, Biru A, Worku A, Desta E, Tafesse H, Dinssa H, et al. Implanon removal experiences of women in Butajira, Ethiopia. The Ethiopian Journal of Health Development. 2015;29(3).
45. Birhane K, Hagos S, Fantahun M. Early discontinuation of implanon and its associated factors among women who ever used implanon in Ofla District, Tigray, Northern Ethiopia. International Journal of Pharma Sciences and Research. 2015;6(3):544-51.
46. Graesslin O, Korver T. The contraceptive efficacy of Implanon®: A review of clinical trials and marketing experience. The European Journal of Contraception & Reproductive Health Care. 2008;13(sup1):4-12.
47. Bhatia P, Nangia S, Aggarwal S, Tewari C. Implanon: subdermal single rod contraceptive implant. The Journal of Obstetrics and Gynecology of India. 2011;61(4):422.
48. Burke H, Ambasa-Shisanya C. Qualitative study of reasons for discontinuation of injectable contraceptives among users and salient reference groups in Kenya. African journal of reproductive health. 2011;15(2).
49. Daff BM, Seck C, Belkhayat H, Sutton P. Informed push distribution of contraceptives in Senegal reduces stockouts and improves quality of family planning services. Global Health: Science and Practice. 2014;2(2):245-52.
50. Trent M, Thompson C, Tomaszewski K. Text messaging support for urban adolescents and young adults using injectable contraception: outcomes of the DepoText pilot trial. Journal of Adolescent Health. 2015;57(1):100-6.
51. Lou C-H, Wang B, Shen Y, Gao E-S. Effects of a community-based sex education and reproductive health service program on contraceptive use of unmarried youths in Shanghai. Journal of Adolescent Health. 2004;34(5):433-40.

For peer review only

BMJ Open

“In fact, that’s when I stopped using contraception”: A qualitative study exploring why sexually active women discontinued contraceptive use in KwaZulu-Natal, South Africa

Journal:	*BMJ Open*
Manuscript ID	bmjopen-2022-063034.R1
Article Type:	Original research
Date Submitted by the Author:	10-Nov-2022
Complete List of Authors:	Hlongwa, Mbuzeleni; University of KwaZulu-Natal College of Health Sciences, Nursing and Public Health<br>Mutambo, Chipo; University of KwaZulu-Natal College of Health Sciences<br>Hlongwana, Khumbulani; University of KwaZulu-Natal College of Health Sciences, Nursing and Public Health; University of KwaZulu-Natal College of Health Sciences, Public Health Medicine
**Primary Subject Heading**:	Reproductive medicine
Secondary Subject Heading:	Public health
Keywords:	Public health < INFECTIOUS DISEASES, Sexual and gender disorders < PSYCHIATRY, REPRODUCTIVE MEDICINE

SCHOLARONE™
Manuscripts

I, the Submitting Author has the right to grant and does grant on behalf of all authors of the Work (as defined in the below author licence), an exclusive licence and/or a non-exclusive licence for contributions from authors who are: i) UK Crown employees; ii) where BMJ has agreed a CC-BY licence shall apply, and/or iii) in accordance with the terms applicable for US Federal Government officers or employees acting as part of their official duties; on a worldwide, perpetual, irrevocable, royalty-free basis to BMJ Publishing Group Ltd ("BMJ") its licensees and where the relevant Journal is co-owned by BMJ to the co-owners of the Journal, to publish the Work in this journal and any other BMJ products and to exploit all rights, as set out in our licence.

The Submitting Author accepts and understands that any supply made under these terms is made by BMJ to the Submitting Author unless you are acting as an employee on behalf of your employer or a postgraduate student of an affiliated institution which is paying any applicable article publishing charge ("APC") for Open Access articles. Where the Submitting Author wishes to make the Work available on an Open Access basis (and intends to pay the relevant APC), the terms of reuse of such Open Access shall be governed by a Creative Commons licence – details of these licences and which Creative Commons licence will apply to this Work are set out in our licence referred to above.

Other than as permitted in any relevant BMJ Author's Self Archiving Policies, I confirm this Work has not been accepted for publication elsewhere, is not being considered for publication elsewhere and does not duplicate material already published. I confirm all authors consent to publication of this Work and authorise the granting of this licence.

For peer review only

"In fact, that's when I stopped using contraception": A qualitative study exploring why sexually active women discontinued contraceptive use in KwaZulu-Natal, South Africa

Mbuzeleni Hlongwa[1, 2], Chipo Mutambo[1], Khumbulani Hlongwana[1]

[1]Discipline of Public Health, School of Nursing and Public Health, University of KwaZulu-Natal, Durban, South Africa.

[2]Burden of Disease Research Unit, South African Medical Research Council, Cape Town, South Africa

Corresponding author:
Mbuzeleni Hlongwa
Discipline of Public Health Medicine,
School of Nursing and Public Health, University of KwaZulu-Natal, Howard College Campus, Glenwood, Durban, 4041, South Africa
hlongwa.mbu@gmail.com

For peer review only - http://bmjopen.bmj.com/site/about/guidelines.xhtml

Abstract

Objectives: This study explored the reasons why sexually active women discontinued contraceptive use in KwaZulu-Natal, South Africa.

Setting: In October 2021, we conducted a qualitative study at Umlazi Township in KwaZulu-Natal province, South Africa, through face-to-face in-depth interviews.

Participants: Fifteen women from four primary health care facilities were recruited through a criterion-based sampling strategy. Using NVivo version 11, two skilled researchers independently conducted thematic data analysis, as a mechanism for quality assurance, before the results were collated and reconciled.

Results: The study included 15 female participants, aged between 18 and 35 years, of whom two-thirds were aged 18-24 years. We found that women were concerned about unpleasant contraceptive side effects such as prolonged or irregular menstrual periods, bleeding, weight gain, and/or severe pains, resulting in discontinuation of their use. In addition to stock outs, women indicated that health care providers did not appropriately counsel or inform them about the available contraceptive methods, including how to use them.

Conclusions: Interventions aimed at reducing contraceptive stock outs are required to ensure that women are empowered to choose contraception based on their own preference, convenience, and/or experience. It is imperative that counselling on contraceptive methods' side effects be improved, to ensure that women have freedom to make informed decisions about their preferred method, proper management of side effects, and to assist them with method switching as needed, instead of discontinuation.

Keywords: Women, experiences, contraceptive use, discontinuation, KwaZulu-Natal, South Africa

For peer review only

Strengths and limitations of this study

- Use of in-depth qualitative interviews elicited rich data that offer important insights into sexually active women's discontinuation of contraceptive use.
- Given that this study focused on women attending public health care facilities, women who do not frequently use public health care clinics may have been excluded, hence their insights would be missing.
- The findings of this study were sought from participants' self-reported sexual and reproductive health information, rendering them prone to social desirability bias.
- Given the sensitivity of the topic, information prone to moral judgements, may have been withheld by the participants for image preservation.

For peer review only

For peer review only - http://bmjopen.bmj.com/site/about/guidelines.xhtml

Introduction

Contraception is one of the most important public health interventions which respond to sexual and reproductive health needs of women, thereby enabling them to plan their pregnancies and decide on the number of children they desire (1). The consistent and correct use of contraception has far-reaching benefits to both individuals and societies, including the reduction of pregnancy-related morbidity and mortality, termination of pregnancies (ToPs), improving educational opportunities, and empowerment of women (2, 3). Furthermore, contraceptive use positively affects the overall health of women of reproductive age, as it empowers them to autonomously make decisions regarding their own sexual and reproductive health (4, 5). Studies have shown that empowered and educated women are more likely to make informed decisions about the use of contraception (6, 7).

Despite the notable improvements in the uptake of contraceptives by women of reproductive age globally, the Sub-Saharan African (SSA) region continues to record a high proportion of women who experience unplanned pregnancies every year (8). In SSA, unmet contraception needs for women of reproductive age continue to be a public health concern, with a substantial number of these women being unable to access and use their preferred methods of contraception, which jeopardizes their desire to circumvent unplanned pregnancies (9, 10). We defined an unmet need for contraception as the proportion of women who are either (a) not pregnant, (b) not postpartum amenorrhoeic, are considered fecund, want to delay their next pregnancy for two years or more, or (c) are postpartum amenorrhoeic and their most recent birth within the previous two years was unwanted or mistimed, but they are not using a contraceptive method (11, 12). The rate of unmet needs for contraceptive use is more than 20% among sexually active women in SSA (13, 14).

The 2016 Demographic and Health Survey report showed that 55% and 60% of married women and sexually active women, respectively, used contraceptives in South Africa (11). At least 15% of women who are in-union, have an unmet need for contraception to prevent unplanned pregnancy, and the figure is slightly higher for sexually active women (19%). However, the KwaZulu-Natal province accounts for 18% and 21% of the national figures of unmet need for contraception, respectively, among in-union and sexually active women (11). In South Africa, more than 100 000 terminations of pregnancy were reported in designated facilities in the

For peer review only - http://bmjopen.bmj.com/site/about/guidelines.xhtml

2016/2017 financial year, with the KwaZulu-Natal accounting for 15% of these (15). Despite ToP being legal in South Africa through the Choice of Termination of Pregnancy Act (CTOPA), which was made public in 1996, and amended in 2008 (16), many ToPs are conducted illegally, contributing to high maternal morbidity and mortality rates (3, 17, 18). The aim of the act is to reduce maternal mortality from illegal termination of pregnancies, and permits pregnant women to terminate their pregnancy legally (16).

High rates of unplanned pregnancy have also been reported among women diagnosed with HIV in South Africa (19, 20). Risky sexual behaviours are also common among young women in this country, as well as in KwaZulu-Natal, including early sexual debut, sex under the influence of alcohol or drugs, multiple sexual partners, and inconsistent condom use, all of which contribute to high levels of unplanned pregnancy and sexually transmitted diseases, including HIV (21-26).

Factors contributing to high rates of unplanned pregnancy in resource-limited settings, including South Africa, are well documented. Insufficient knowledge of contraception, gender inequality, intimate partner violence, poverty, and inconsistent and incorrect use of contraception, are some of the factors (8, 27-29). Low levels of contraceptive use have been associated with age, low education level and low socioeconomic status, limited knowledge and inaccessibility of contraceptive methods, and resource-limited rural residential settings (23, 29-34). Among adolescent girls, some of the barriers to contraceptive use include the lack of desire to avoid, delay, space or limit childbearing; the lack of confidence and ability to seek and/or negotiate contraceptive use; the lack of desire to use contraception; and poor access to contraceptive services and to quality (35). Health system challenges, including contraception stock-outs, long waiting times, and negative attitudes displayed by some health care providers, have also been reported to deter women, especially young women, from accessing contraceptive services from health care facilities, thereby contributing to low or inconsistent uptake of contraceptives (33, 36-38). High rates (29%) of contraceptive users discontinue using contraception within a year of starting to use contraception, due to side effects (28%), the desire to become pregnant (19%) or the desire for a more effective method (11%) (11). Contraceptive stockouts remain high in South Africa, deterring women from accessing preferred contraceptive methods in local public health care facilities, thereby contributing to low or inconsistent uptake of contraceptives (11, 39, 40).

For peer review only - http://bmjopen.bmj.com/site/about/guidelines.xhtml

Despite these challenges, the South African government has demonstrated commitment to ensuring universal access to contraception by women of reproductive age. In South Africa, only 46% of births occurring within a five-year period preceding the SADHS 2016, were intended at the conception time, while 34% and 20%, respectively, were mistimed and unwanted (11). This study explored the reasons why sexually active women discontinued contraceptive use in KwaZulu-Natal, South Africa. The use of qualitative research methods deepened our understanding of this phenomenon.

Methods

Study setting

Umlazi, a township populated with more than half a million people, is located in the province of KwaZulu-Natal (41) and is part of the eThekwini Metropolitan Municipality, which has the largest number of people on lifelong anteretroviral therapy (ART) in the province (42). Umlazi has 10 public health care clinics and one public health hospital. Four public health care clinics, spread across various parts of Umlazi, participated in the study.

Study design

We explored why sexually active women discontinued contraceptive use, using an exploratory descriptive qualitative study design (43). We generated data in October 2021, through face-to-face in-depth interviews with women of reproductive age accessing health care services, from four facilities in Umlazi.

Sampling

We employed criterion-based sampling strategies, to identify potentially eligible women from four primary health care facilities. In order to be eligible for selection, women had to meet the following criteria: (a) be of reproductive age (18–49 years); (b) reside in Umlazi Township; (c) be sexually active (women who had sexual intercourse within three months preceding data collection); and (d) use contraception or would have used contraception within three months preceding data collection. Women who were outside the age brackets (18-49 years), pregnant, or sexually inactive, were excluded from the study. Interviews were conducted after services had been rendered by the facility.

For peer review only

Data collection tool

We developed an in-depth interview guide in the English language and then translated it into IsiZulu, which is the dominant language used in Umlazi. A female research assistant with a track record of conducting interviews and qualitative research, was recruited and provided with refresher training prior to collecting the data. The research assistant is fluent in both English and IsiZulu languages. Interviews were audio-recorded (with participants' permission). To minimize any inconsistencies during the data collection period, the interview guide was pre-tested with five participants who did not form part of the study. The scope of inquiry of the interviews focused on four key components of contraception, namely: (i) awareness about different contraceptive methods, (ii) access (availability/stock-outs of preferred contraceptive methods, counselling), (iii) uptake (key considerations in deciding whether or not to start using contraception), and (iv) adherence/continuation (key considerations in deciding whether or not to continue or discontinue using contraception, including side effects, violence, stigma, discrimination, judgements).

Data collection

Data was collected iteratively to ensure that researchers engaged with preliminary data analysis of the information collected, learn emerging themes, identify gaps in the data, and adapt the data collection process for subsequent interviews. Each interview lasted for 30 to 60 minutes. Data saturation was reached at 15 interviews. At least five potential participants refused to participate in the study when they were approached. The lead author held regular debriefing meetings with the research assistant to discuss field experiences, challenges, emergent issues, lessons learned, and how she affected and/or was affected by the interviews. Data collection only took place after the services had been rendered by the facility. These participants were recruited regardless of the service they presented themselves for at the facility. We did not collect information regarding the care service they visited the facility for.

Data analysis

Using NVivo version 11, two researchers independently conducted the analysis from data coding to the development of themes, and this was done iteratively, guided by Richie and Spencer's framework (44). The framework outlines the following stages for conducting qualitative data analysis: (a) familiarisation with the data through reading all the transcripts and listening to the audio recordings; (b) generating initial codes using an open coding method,

For peer review only - http://bmjopen.bmj.com/site/about/guidelines.xhtml

where each segment of data relevant to this study's research objective was coded; (c) development of a thematic framework extracting key themes from the coded data; (d) application of the thematic framework to all the data; (e) charting of the data, enabling systematic comparisons between data sets; and (f) analysis of the charts for patterns and associations between and within each unit of analysis.

Data quality

To ensure rigor and accuracy, comparative data analysis was conducted by two skilled researchers, who independently read all the transcripts to gain an understanding of the content and scope of the data collected, prior to data coding. The outcome of coding was verified, cross-checked, and thoroughly discussed between the two members, to ensure that the research question was answered. We used the COREQ checklist to ensure that the study adhered to quality standards for reporting qualitative study findings (45).

Ethical considerations

Ethics approval and gatekeeper permission were obtained from the University of KwaZulu-Natal Biomedical Research Ethical Committee (BREC) (Ref No: BE424/18) and the Department of Health's National Health Research Database (NHRD) (Ref No: KZ_2018009_013), respectively. The eThekwini District's Ethical Review Committee also approved the study. The study was also supported by the participating health facilities. All participants who volunteered to participate in the study signed an informed consent form prior to their participation. The privacy and confidentiality of participants were protected.

Patient and Public Involvement

Patients or the public were not involved in the design, or conduct, or reporting, or dissemination plans of our research

Results

Background characteristics of study participants

The study included 15 women, aged between 18 and 35 years, with a median age of 23 years (Table 1). All participants were Black African women, with more than two-thirds (n=10) aged 18-24 years. The majority (n=12) had attained a secondary level of education and nearly half (n=7) were unemployed. Almost all participants (n=14) were not married.

For peer review only - http://bmjopen.bmj.com/site/about/guidelines.xhtml

Table 1: Demographic characteristics of participants, Umlazi Township, KwaZulu-Natal, South Africa, 2021

Characteristic	Participants n=15
Age (years)	18-49
Median (IQR)	23 (19-26)
Age categories, n (%)	
18-24 years	10 (67)
25-49 years	5 (33)
Population group, n (%)	
Black African	15 (100)
Level of education, n (%)	
Secondary	12 (80)
Tertiary	3 (20)
Employment status, n (%)	
Unemployed	7 (47)
Employed	4 (27)
Studying	4 (27)
Marital status, n (%)	
Not married	14 (93)
Married	1 (7)

Key themes

The following key themes were developed during analysis: concerns over contraceptives' side effects; contraceptive methods' stock-outs, inconsistent or incorrect use of contraceptive methods; inadequate counselling on contraceptive methods; and misconceptions about contraception.

Concerns over contraceptives' side effects

In addition to contraceptive mistrust, there were reports of unpleasant contraceptive side effects from some of the participants. The most common side effect reported by the participants, was prolonged menstrual periods resulting from using injectable contraceptives.

"The injection made me have prolonged periods than usual. I usual go on periods for three days, but after taking the injection my periods would last for seven days… I was tired of being on periods all the time. My periods lasted longer than usual. I'd keep having periods. Then it would stop eventually, but then again the following months when it's time for my periods, they would last longer… The next month, same thing". (19-year-old)

"I went on a prolonged menstrual cycle, my periods started from the 2nd to the 12th August… I don't know what is going on, I don't know…But the injection didn't treat me well. I had

For peer review only - http://bmjopen.bmj.com/site/about/guidelines.xhtml

started with the two months injection. But I stopped using the two months injection because it would make me go on periods at any time. It made me bleed at any time". (19-year-old)

Some women indicated that using the injectable contraceptives made them bleed heavily, or that the injectable interfered with their menstrual cycle, and resulted in inconsistent menstrual periods.

She [nurse] had given me the 3 months injection. The three months injection was making me bleed a lot. My bleeding couldn't stop. Then I did some research and asked from others and they said that it happens that the injection would not respond well on your body". (20-year-old)

"It was not treating me well. It would make me go on periods at least twice in one month. I would go on periods for three or four days but twice a month. In the beginning it treated me well. Then it changed later on". (25-year-old)

Women reported that using contraception contributed to changes in their appetite for food, thereby affecting their weight. Some experienced weight gain and others lost weight.

"I would eat a lot. I would eat every now and then. And if I wanted food, I really wanted food, like someone who's pregnant". (20-year-old)

"...when I started with the contraceptives, I gained a lot of weight and I was oily, like my face was messed up so I stopped and then after a year I went back [re-started contraception]...". (22-year-old)

"I started by using the three months injection, but it made me lose weight so bad, then I switched from it to the two months injection". (25-year-old)

Some women indicated that they experienced severe pains, as a result of using the injectable contraceptive method.

"No, but I've used injection before and the injection didn't treat me well. I always had problems in my bladder and I decided to stop using it. I would have unusual pains, which is something I had never experienced before I started using contraception". (27-year-old)

Some participants reacted to side effects by simply discontinuing contraception.

For peer review only - http://bmjopen.bmj.com/site/about/guidelines.xhtml

"The injection made me have [more] extended periods than usual… So I didn't even ask, I just decided to stop using contraception. In fact, that's when I stopped using contraception. I stopped using contraception last year or last-of-last year". (19-year-old)

"At the clinic they told me there's this kind of injection and I should try it. But I then stopped it when I experienced these side effects. I would also get sick. I was supposed to go on periods. But because of the injection I wouldn't go on periods. I stopped it". (27-year-old)

Others reacted to side effects by simply switching to a different contraceptive method.

"Then I came back and told the nurse about the side effects I was experiencing while using that injection. I then asked her to switch me to a different method. And then she switched me to a two-months injection". (20-year-old)

"I told them that I'd like to switch from using this contraception because I had started using the pills for a short while. So I knew how they worked. Then I asked them to change me to the pills. Now I only go on periods once a month". (35-year-old)

Stock-outs of preferred contraceptive methods and participants' reactions

Participants reported stock-outs of their preferred contraceptive methods as a pervasive challenge in the health care facilities.

"Yes, even last week they said they were out of stock, especially the three months injection, they only had the two month's one". (18-year-old)

"…sometimes you come here and find that injection is not available… I don't know what can they do to improve the situation. I sometimes ask nurses why they don't have the injections and they say that it is the department that is not delivering them". (20-year-old)

One participant saw Implanon as a potential solution to the challenge of stock-outs, as this method does not require her to make regular visits to the facility for contraceptive purposes.

"…so, I'm considering using it [Implanon] because the injection is sometimes not available at the clinic. And they said at the clinic that if the injections are finished again, they don't know when it will be available again…". (23-year-old)

For peer review only - http://bmjopen.bmj.com/site/about/guidelines.xhtml

"When I got to this clinic, I was told that injectable contraceptive methods is no longer available. They told me that there is the implant contraceptive method. But I'm scared of implant. I ended up stopping using contraception because I was scared of using the implant. I'm still going to think about the implant but it is scary…I came [to the clinic] on September 15, and I asked for implant and they said that I should come back another day….They do tell you that it's not that they don't offer it, it's just out of stock, you should come back". (24-year-old)

Changing to an alternative contraception method was one of the options pursued by some participants whenever they were unable to receive their preferred contraceptive method, while others opted to wait for their preferred contraception. Those who opted to wait for their contraception of choice, had to modify their lifestyle during the period of being not protected by contraception.

"I continued using the three months injection. But it would happen that sometimes they would not have it at the clinic. Then I would be switched to the two months injection". (20-year-old)

"I decided to stop. I didn't take them. I stopped using contraceptives. My date was on the 12th September. I came here and I was told that the injection is not available. Then, again I came back on the 18th September. And they told me again that the injection is not available. Then I waited. Then I received a message from my friend who told me that she was at the clinic and that the injection is now available. I think it was last week. Then I came back and took the injection". (20-year-old)

"For me I don't want to use something else when I'm still going to use the implant. I don't want too many in my body. At least if I use one. I should not mix. I should just be patient for the implant. I should be able to control myself. For now, I'm trying to avoid having sex, since August". (24-year-old)

The contraception stock-outs was reported to have far-reaching implications on women, as it does not only make them vulnerable to unplanned pregnancies, but also causes emotional discomfort.

"During that time the injection is not available, you are not comfortable. You are scared. It is hard when the injection is not available". (20-year-old)

Others were discouraged to continue using contraception due to stock-outs.

For peer review only - http://bmjopen.bmj.com/site/about/guidelines.xhtml

"You'll end up getting discouraged if you don't get the contraceptive methods you need. All they need to do is to ensure that the preferred contraceptive methods are always available". (35-year-old)

Inconsistent or incorrect use of contraceptive methods

Participants indicated that they sometimes could not consistently use contraceptives, as some of them missed their re-injection appointments. This resulted in some of them experiencing an unplanned pregnancy.

"…When my re-injection date had passed, as I was taking a break, so that my blood will flow and the side effects would stop, I then got pregnant. I had planned to wait for a month. Then I would start the following month. It was stupid I don't know how [laughs]. And then during that period I became pregnant. It just happened so quickly. I couldn't believe it. I couldn't believe that I was really pregnant". (23-year-old)

"I do have a child. I didn't stop using contraception, but I got the child after I skipped my date for re-injection. It just happened that I got pregnant". (25-year-old)

Most participants showed less preference for the contraceptive pills, fearing that they would not be able to maintain or use them consistently or correctly.

"…what made me stop using them [contraceptive pills] is because I used to miss them because you have to take them at the same time daily, I stopped taking them… I stopped using them because I kept missing the time whereas you are supposed to take them at the same time". (19-year-old)

"I've never used something else. I've only used the injection. Because I cannot use the pills because I would forget them. I forget the pills I cannot use them". (23-year-old)

Some participants reported instances where health care providers either inserted the implant incorrectly, or inserted it into a pregnant woman, as they hardly ever checked a woman's pregnancy status before inserting the implant.

"…and I also got pregnant while the implant was inserted in me… Maybe they inserted it while I was already pregnant. They didn't check my pregnancy status. They didn't run pregnancy tests. They just inserted it". (19-year-old)

For peer review only - http://bmjopen.bmj.com/site/about/guidelines.xhtml

"I had pregnancy symptoms. I would vomit. I would experience things that I didn't understand. Then I went back to the clinic and I was told that the implant was not inserted correctly. Then they started telling me about the possible risks since I am already pregnant. That I might get a miscarriage. And I did get a miscarriage. I don't know if that's what caused the miscarriage. After they removed the implant, I had already lost hope because I was bleeding". (19-year-old)

Lack of counselling on contraceptive methods

Participants indicated that they were not counselled or given information regarding the use of, and the possible side effects, of various contraceptive methods offered at health care facilities.

"…the nurse did not explain anything to me. She did not tell me how it works. I do have some questions about using contraception. I want to ask that since they are different, there's one for three months and another one for two months – what's the difference between them? That's all I want to ask". (18-year-old)

"She didn't even ask me which method I wanted to use or what. She didn't and I couldn't ask questions. I didn't even know which injection she was giving to me [after giving birth at the hospital]. She didn't say anything. She just calls your name and then gives you the injection….. It got me confused. Because I wanted to use a different method. But I only realized what they had given me when I was reading the card because it was written there. And there was nothing I could still do". (20-year-old)

"…there was no opportunity because in the room we were getting injections from, we were many so there was no space for discussing or asking questions. So you'd get the injection and go home. You didn't have time to discuss things or ask questions". (24-year-old)

Misconceptions about contraception

Concerns over the effectiveness of using contraceptive methods were raised, with some women reporting that they do not trust that the contraceptives are effective enough to prevent someone from getting an unplanned pregnancy.

"I think that because there's many different contraceptive methods, what I know about the injection is that it reduces chances of getting pregnant. But it is not 100% accurate…. I will continue using a condom because I know that the injection is not 100% sure." (18-year-old)

For peer review only - http://bmjopen.bmj.com/site/about/guidelines.xhtml

Some participants raised concerns about the possibility of contraception impairing future fertility, with others preferring to wait until they got their first child before using contraceptive method.

"…but the very first time I heard about contraception, I was told by my mother who asked me to use contraception, so that I won't get pregnant. I hadn't had a child at that time. But I refused because I heard people saying that if you use contraception when you haven't had a child, it happens that you might not be able to conceive. So I didn't use contraception. I only started using contraception after getting a baby. I have one child". (20-year-old)

"I only used it for a short period because I was told that it's dangerous to use the injection when you don't have a child because it completely prevents you from getting pregnant in future….". (25-year-old)

"I do have questions. I have many questions. Because even now I am concerned. Some people do say that the injection makes you infertile even after you have stopped using it. You won't go on periods. Now that it's been months without seeing my periods, I'm scared". (19-year-old)

Discussion

In this study, we adopted a qualitative approach to exploring reasons for contraception discontinuation among sexually active women in KwaZulu-Natal, South Africa. Our study participants shared crucial insights for understanding the challenges women face, when accessing and using contraceptive methods in Umlazi Township, KwaZulu-Natal. Our results show that concerns over contraceptives' side effects; contraceptive methods' stock-outs, inconsistent or incorrect use of contraceptive methods; inadequate counselling on contraceptive methods; and misconceptions about contraception, affect the use of contraceptives in the study setting, and this may have some relevance for other comparable settings.

Side effects play an important role in a woman's decision as to whether to continue or discontinue using contraception, as has been reported in other studies (46, 47). Similar to our study, prolonged or irregular menstrual cycle patterns have also been reported as one of the most common side effects that influence women to discontinue contraceptive use (47-49). Both the injectable users and implant users have concerns about bleeding side effects, which are

For peer review only - http://bmjopen.bmj.com/site/about/guidelines.xhtml

known to have contributed to discontinuation of contraception in similar settings (47). Contraceptive discontinuation has dire implications, including unplanned pregnancy. Therefore, women should be empowered to avoid unplanned pregnancies, by identifying alternative contraceptive methods that meet their individual needs.

There has been concerns about the growing number of women who return to clinics for implant removal, only after a few months of insertion, due to changes in bleeding patterns or excessive bleeding (48, 50, 51). Side effects, notably prolonged or irregular bleeding, have also been reported by health care providers as the most common reason for early Implanon discontinuation (48). In addition to this, other factors have been reported to influence women to remove the implant, including incorrect positioning and low quality of care (52-54). Incorrect positioning of the implant was also reported in this study as an important factor for its discontinuation. The implant protects against pregnancy for up to three years before it needs to be replaced, and this reduces frequent clinic visits among users (55). However, the growing number of women removing the implant given the side effects, suggests the need for pre-insertion counselling, proper management, and empowerment of women through information regarding alternative contraceptive options for better decision-making (51).

Contraceptive stock-outs occur when one or more contraceptive methods are not available at a health care facility that routinely provides that method. Contraception stock-outs are one of the main factors contributing to contraceptive switching and discontinuation among women (56, 57). Contraceptive methods should be available at all levels of health care, but not all health care facilities offer the full mix of modern contraceptive methods. Women have different contraceptive method preferences and experiences, therefore, a wide variety of contraceptive methods should always be available; this ensures that women are empowered to choose contraception based on their own preference, convenience, and/or experience. It has been reported that the use of modern contraception increases when more methods become available (58). Given the high rates of contraceptive method stock-outs in South Africa, unemployed women are likely to suffer the most, due to financial constraints, whereas women with financial resources, may be able to access preferred contraceptive methods through the private sector.

For peer review only - http://bmjopen.bmj.com/site/about/guidelines.xhtml

Health care providers play an important role in ensuring access to contraceptive methods among women. As such, health care providers have an important responsibility in educating women and providing complete information about possible side effects, and the effectiveness of their preferred method. It is imperative that counselling on side effects be improved, to ensure that women have freedom to make informed decisions about their preferred method, and to assist them with method switching as needed. However, counselling when receiving family planning services is limited in South Africa (40). Moreover, a single face-to-face counselling appointment in a busy facility may not be enough to convey all of the information a woman requires about: the reproductive cycle, returning to fertility after discontinuing a method, potential drug-drug interactions, the need for dual protection, and so on (40). In addition to traditional face-to-face interactions between health care providers and contraceptive users, innovative approaches, such as internet-based sources of information, text message reminders, and brochures are needed to improve women's comprehension of how contraceptive methods work (40, 59, 60).

Our study has important limitations to note. The findings of this study were sought from participants' self-reported sexual and reproductive health information, rendering them prone to social desirability bias. Given the sensitivity of the topic, information deemed to have the potential for leading to judgements, may have been withheld by the participants for image preservation. Given that this study focused on women attending public health care facilities, women who do not frequently use public health care clinics may have been excluded, hence their insights would be missing. Lastly, due to the qualitative nature of the study, the fact that this study was confined to limited health care facilities and participants, meant that our study findings cannot be generalised to other settings. However, the study provides important insights regarding the perceptions and experiences of contraceptive use among sexually active women in Umlazi Township, KwaZulu-Natal, South Africa.

Conclusion

Our findings illustrate that concerted efforts are urgently required to address women's concerns regarding the side effects arising from using contraceptive methods, as well as contraceptive stock-outs, given the dire implications these may have on contraceptive discontinuation and subsequent unplanned pregnancy. The provision of comprehensive counselling services to support women who are having short-term side effects is paramount, to ensure that they are

For peer review only - http://bmjopen.bmj.com/site/about/guidelines.xhtml

able to deal with side effects, or switch to a different method instead of completely discontinuing contraceptive use, to avoid unplanned pregnancy.

Declarations

Ethics approval and consent to participate: Ethics approval was obtained through the Biomedical Research Ethics Committee from the University of KwaZulu-Natal (Ref No: BE424/18). Approval was obtained through the National Health Research Database from the KwaZulu-Natal Provincial Department of Health (Ref No: KZ_2018009_013).

Consent for publication: Not applicable.

Availability of data and materials: All the data analysed and reported in this paper will be made available upon request.

Competing interest: The authors declare that they have no competing interests.

Funding: The work reported herein was made possible through funding by the South African Medical Research Council through its Division of Research Capacity Development under the **BONGANI MAYOSI NATIONAL HEALTH SCHOLARS PROGRAM** from funding received from the South African National Treasury. The content hereof is the sole responsibility of the authors and does not necessarily represent the official views of the SAMRC or the funders.

Authors' contributions: MH conceptualized and designed the study, as well as prepared the initial draft. KH and CM reviewed the manuscript. All the authors approved the final version of the manuscript.

Acknowledgements: The authors would like to thank the School of Nursing and Public Health, University of KwaZulu-Natal, Durban, South Africa and the Division of Research Capacity Development at SA MRC.

For peer review only

References

1. Bongaarts J, Cleland JC, Townsend J, Bertrand JT, Gupta MD. Family planning programs for the 21st century: rationale and design. 2012.
2. Michalow J, Chola L, McGee S, Tugendhaft A, Pattinson R, Kerber K, et al. Triple return on investment: the cost and impact of 13 interventions that could prevent stillbirths and save the lives of mothers and babies in South Africa. BMC Pregnancy and Childbirth 2015;15(1):1-13.
3. Darroch JE, Woog V, Bankole A, Ashford LS. Adding it up: costs and benefits of meeting the contraceptive needs of adolescents. 2016.
4. Ganatra B, Faundes A. Role of birth spacing, family planning services, safe abortion services and post-abortion care in reducing maternal mortality. Best Practice & Research Clinical Obstetrics & Gynaecology. 2016;36:145-55.
5. Hameed W, Azmat SK, Ali M, Sheikh MI, Abbas G, Temmerman M, et al. Women's empowerment and contraceptive use: the role of independent versus couples' decision-making, from a lower middle income country perspective. PloS one. 2014;9(8):e104633.
6. Corroon M, Speizer IS, Fotso J-C, Akiode A, Saad A, Calhoun L, et al. The role of gender empowerment on reproductive health outcomes in urban Nigeria. Maternal and child health journal. 2014;18(1):307-15.
7. Tekelab T, Melka AS, Wirtu D. Predictors of modern contraceptive methods use among married women of reproductive age groups in Western Ethiopia: a community based cross-sectional study. BMC women's health. 2015;15(1):1-8.
8. Cleland J, Harbison S, Shah IH. Unmet need for contraception: issues and challenges. Studies in family planning. 2014;45(2):105-22.
9. United Nations. Family Planning and the 2030 Agenda for Sustainable Development. New York: United Nations, Department of Economic and Social Affairs, Population Division.; 2019.
10. Chersich MF, Wabiri N, Risher K, Shisana O, Celentano D, Rehle T et al. Contraception coverage and methods used among women in South Africa: A national household survey. South African Medical Journal 2017;107(4):307-14.
11. NDoH, Stats SA, SAMRC, ICF. South Africa Demographic and Health Survey 2016. Pretoria, South Africa, and Rockville, Maryland, USA: National Department of Health (NDoH), Statistics South Africa (Stats SA), South African Medical Research Council (SAMRC), ICF; 2019.
12. Sedgh G, Ashford LS, Hussain R. Unmet need for contraception in developing countries: examining women’s reasons for not using a method. 2016.
13. Darroch JE, Sully E, Biddlecom A. Adding it up: investing in contraception and maternal and newborn health, 2017—supplementary tables. New York, NY: The Guttmacher Institute. 2017.
14. World Health Organization. Contraception. Geneva, Switzerland: World Health Organization; 2019.
15. SA National Department of Health. Pregancy terminations in designated South African facilities. Pretoria: SA National Department of Health; 2017.
16. Republic of South Africa. Choice on Termination of Pregnancy Act (No. 92 of 1996). Government Gazette. 1996;377(17602).
17. Hodes R. The culture of illegal abortion in South Africa. Journal of Southern African Studies. 2016;42(1):79-93.
18. Seidu A-A, Ahinkorah BO, Agbemavi W, Amu H, Bonsu F. Reproductive health decision-making capacity and pregnancy termination among Ghanaian women: Analysis of the 2014 Ghana demographic and health survey. Journal of Public Health. 2021;29(1):85-94.
19. Adeniyi OV, Ajayi AI, Moyaki MG, Ter Goon D, Avramovic G, Lambert J. High rate of unplanned pregnancy in the context of integrated family planning and HIV care services in South Africa. BMC health services research. 2018;18(1):1-8.

For peer review only - http://bmjopen.bmj.com/site/about/guidelines.xhtml

20. Schwartz SR, Rees H, Mehta S, Venter WDF, Taha TE, Black V. High incidence of unplanned pregnancy after antiretroviral therapy initiation: findings from a prospective cohort study in South Africa. PloS one. 2012;7(4):e36039.
21. Hlongwa M, Peltzer K, Hlongwana K. Risky sexual behaviours among women of reproductive age in a high HIV burdened township in KwaZulu-Natal, South Africa. BMC Infectious Diseases. 2020;20(1):1-9.
22. Toska E, Cluver LD, Boyes M, Pantelic M, Kuo C. From 'sugar daddies' to 'sugar babies': exploring a pathway among age-disparate sexual relationships, condom use and adolescent pregnancy in South Africa. Sexual health. 2015;12(1):59-66.
23. Christofides NJ, Jewkes RK, Dunkle KL, Nduna M, Shai NJ, Sterk C. Early adolescent pregnancy increases risk of incident HIV infection in the Eastern Cape, South Africa: a longitudinal study. Journal of the International AIDS Society. 2014;17(1):18585.
24. Atuyambe LM, Kibira SP, Bukenya J, Muhumuza C, Apolot RR, Mulogo E. Understanding sexual and reproductive health needs of adolescents: evidence from a formative evaluation in Wakiso district, Uganda. Reproductive health. 2015;12(1):1-10.
25. Solanke BL. Factors influencing contraceptive use and non-use among women of advanced reproductive age in Nigeria. Journal of Health, Population and Nutrition. 2017;36(1):1-14.
26. Blackstone SR, Iwelunmor J. Determinants of contraceptive use among Nigerian couples: evidence from the 2013 Demographic and Health Survey. Contraception and reproductive medicine. 2017;2(1):1-8.
27. Maharaj P. Stalling contraception? Perspectives and experiences of sexually active women and men. Agenda. 2012;26(2):100-10.
28. Jewkes R, Morrell R, Christofides N. Empowering teenagers to prevent pregnancy: lessons from South Africa. Culture, health & sexuality. 2009;11(7):675-88.
29. Chersich MF, Wabiri N, Risher K, Shisana O, Celentano D, Rehle T, et al. Contraception coverage and methods used among women in South Africa: A national household survey. South African Medical Journal 2017;107(4):307-14
30. Makola L, Mlangeni L, Mabaso M, Chibi B, Sokhela Z, Silimfe Z, et al. Predictors of contraceptive use among adolescent girls and young women (AGYW) aged 15 to 24 years in South Africa: results from the 2012 national population-based household survey. BMC women's health. 2019;19(1):1-7.
31. Digitale J, Psaki S, Soler-Hampejsek E, Mensch BS. Correlates of contraceptive use and health facility choice among young women in Malawi. The ANNALS of the American Academy of Political and Social Science. 2017;669(1):93-124.
32. Nyarko SH. Prevalence and correlates of contraceptive use among female adolescents in Ghana. BMC women's health. 2015;15(1):1-6.
33. Hlongwa M, Mashamba-Thompson T, Makhunga S, Hlongwana K. Evidence on factors influencing contraceptive use and sexual behavior among women in South Africa: A scoping review. Medicine. 2020;99(12):e19490.
34. Hlongwa M, Kalinda C, Peltzer K, Hlongwana K. Factors associated with modern contraceptive use: a comparative analysis between younger and older women in Umlazi Township, KwaZulu-Natal, South Africa. Women's Health. 2021;17:17455065211060641.
35. Chandra-Mouli V, Akwara E. Improving access to and use of contraception by adolescents: what progress has been made, what lessons have been learnt, and what are the implications for action? Best Practice & Research Clinical Obstetrics & Gynaecology. 2020;66:107-18.
36. Hlongwa M, Tlou B, Hlongwana K. Healthcare providers' knowledge and perceptions regarding the use of modern contraceptives among adolescent girls in Umlazi Township, KwaZulu-Natal province, South Africa. The Pan African Medical Journal. 2021;38.
37. Smith P, Marcus R, Bennie T, Nkala B, Nchabeleng M, Latka M, et al. What do South African adolescents want in a sexual health service? Evidence from the South African Studies on HIV in Adolescents (SASHA) project. South African Medical Journal. 2018;108(8).

For peer review only - http://bmjopen.bmj.com/site/about/guidelines.xhtml

38. Jonas K, Duby Z, Maruping K, Dietrich J, Slingers N, Harries J, et al. Perceptions of contraception services among recipients of a combination HIV-prevention interventions for adolescent girls and young women in South Africa: a qualitative study. Reproductive Health. 2020;17(1):1-14.
39. Blanc AK, Curtis SL, Croft TN. Monitoring contraceptive continuation: links to fertility outcomes and quality of care. Studies in family planning. 2002;33(2):127-40.
40. Lince-Deroche N, Pleaner M, Morroni C, Mullick S, Firnhaber C, Harries J, et al. Achieving universal access to sexual and reproductive health services: the potential and pitfalls for contraceptive services in South Africa. South African Health Review. 2016;2016(1):95-108.
41. Statistics South Africa. Census 2011. Pretoria: Statistics South Africa; 2012.
42. Simbayi LC, Zuma K, Zungu N, Moyo S, Marinda E, Jooste S, et al. South African National HIV Prevalence, Incidence, Behaviour and Communication Survey, 2017. Cape Town: HSRC; 2019.
43. Parker I. Discourse analysis: Dimensions of critique in psychology. Qualitative Research in Psychology. 2013;10(3):223-39.
44. Ritchie J, Spencer L. Qualitative data analysis for applied policy research. Analyzing qualitative data: Routledge; 2002. p. 187-208.
45. Tong A, Sainsbury P, Craig J. Consolidated criteria for reporting qualitative research (COREQ): a 32-item checklist for interviews and focus groups. International journal for quality in health care. 2007;19(6):349-57.
46. Castle S, Askew I. Contraceptive discontinuation: Reasons, challenges and solutions: Population Council. FP2020. 2015.
47. Keogh SC, Otupiri E, Castillo PW, Chiu DW, Polis CB, Nakua EK, et al. Hormonal contraceptive use in Ghana: the role of method attributes and side effects in method choice and continuation. Contraception. 2021.
48. Adeagbo O, Mullick S, Pillay D, Chersich M, Morroni C, Naidoo N, et al. Uptake and early removals of Implanon NXT in South Africa: Perceptions and attitudes of healthcare workers. South African Medical Journal. 2017;107(10).
49. Veisi F, Zangeneh M. Comparison of two different injectable contraceptive methods: Depo-medroxy progesterone acetate (DMPA) and cyclofem. Journal of family & reproductive health. 2013;7(3):109.
50. Burusie A. Reasons for premature removal of Implanon among users in Arsi zone, Oromia region, Ethiopia, 2013. Reproductive System & Sexual Disorders: Current Research. 2015;4(1):1-6.
51. Mrwebi KP, Ter Goon D, Owolabi EO, Adeniyi OV, Seekoe E, Ajayi AI. Reasons for discontinuation of Implanon among users in Buffalo City Metropolitan Municipality, South Africa: a cross-sectional study. African journal of reproductive health. 2018;22(1):113-9.
52. Zerihun H, Biru A, Worku A, Desta E, Tafesse H, Dinssa H, et al. Implanon removal experiences of women in Butajira, Ethiopia. The Ethiopian Journal of Health Development. 2015;29(3).
53. Birhane K, Hagos S, Fantahun M. Early discontinuation of implanon and its associated factors among women who ever used implanon in Ofla District, Tigray, Northern Ethiopia. International Journal of Pharma Sciences and Research. 2015;6(3):544-51.
54. Graesslin O, Korver T. The contraceptive efficacy of Implanon®: A review of clinical trials and marketing experience. The European Journal of Contraception & Reproductive Health Care. 2008;13(sup1):4-12.
55. Bhatia P, Nangia S, Aggarwal S, Tewari C. Implanon: subdermal single rod contraceptive implant. The Journal of Obstetrics and Gynecology of India. 2011;61(4):422.
56. Burke H, Ambasa-Shisanya C. Qualitative study of reasons for discontinuation of injectable contraceptives among users and salient reference groups in Kenya. African journal of reproductive health. 2011;15(2).

For peer review only - http://bmjopen.bmj.com/site/about/guidelines.xhtml

57. Daff BM, Seck C, Belkhayat H, Sutton P. Informed push distribution of contraceptives in Senegal reduces stockouts and improves quality of family planning services. Global Health: Science and Practice. 2014;2(2):245-52.
58. Ross J, Stover J. Use of modern contraception increases when more methods become available: analysis of evidence from 1982–2009. Global Health: Science and Practice. 2013;1(2):203-12.
59. Trent M, Thompson C, Tomaszewski K. Text messaging support for urban adolescents and young adults using injectable contraception: outcomes of the DepoText pilot trial. Journal of Adolescent Health. 2015;57(1):100-6.
60. Lou C-H, Wang B, Shen Y, Gao E-S. Effects of a community-based sex education and reproductive health service program on contraceptive use of unmarried youths in Shanghai. Journal of Adolescent Health. 2004;34(5):433-40.

For peer review only

For peer review only - http://bmjopen.bmj.com/site/about/guidelines.xhtml

Standards for Reporting Qualitative Research: A Synthesis of Recommendations

TOPIC	NO.	ITEM	REPORTED ON PAGE #
**TITLE**			
Title	S1	Concise description of the nature and topic of the study Identifying the study as qualitative or indicating the approach (e.g., ethnography, grounded theory) or data collection methods (e.g., interview, focus group) is recommended	1
Abstract	S2	Summary of key elements of the study using the abstract format of the intended publication; typically includes background, purpose, methods, results, and conclusions	2
**INTRODUCTION**			
Problem formulation	S3	Description and significance of the problem/phenomenon studied; review of relevant theory and empirical work; problem statement	4-5
Purpose or research question	S4	Purpose of the study and specific objectives or questions	6
**METHODS**			
Qualitative approach and research paradigm	S5	Qualitative approach (e.g., ethnography, grounded theory, case study, phenomenology, narrative research) and guiding theory if appropriate; identifying the research paradigm (e.g., postpositivist, constructivist/ interpretivist) is also recommended; rationale [b]	6
Researcher characteristics and reflexivity	S6	Researchers' characteristics that may influence the research, including personal attributes, qualifications/experience, relationship with participants, assumptions, and/or presuppositions; potential or actual interaction between researchers' characteristics and the research questions, approach, methods, results, and/or transferability	6-7
Context	S7	Setting/site and salient contextual factors; rationale [b]	6
Sampling strategy	S8	How and why research participants, documents, or events were selected; criteria for deciding when no further sampling was necessary (e.g., sampling saturation); rationale [b]	6
Ethical issues pertaining to human subjects	S9	Documentation of approval by an appropriate ethics review board and participant consent, or explanation for lack thereof; other confidentiality and data security issues	8
Data collection methods	S10	Types of data collected; details of data collection procedures including (as appropriate) start and stop dates of data collection and analysis, iterative process, triangulation of sources/methods, and modification of procedures in response to evolving study findings; rationale [b]	7
Data collection instruments and technologies	S11	Description of instruments (e.g., interview guides, questionnaires) and devices (e.g., audio recorders) used for data collection; if/how the instrument(s) changed over the course of the study	7

For peer review only

TOPIC	NO.	ITEM	REPORTED ON PAGE #
Units of study	S12	Number and relevant characteristics of participants, documents, or events included in the study; level of participation (could be reported in results)	9
Data processing	S13	Methods for processing data prior to and during analysis, including transcription, data entry, data management and security, verification of data integrity, data coding, and anonymization/deidentification of excerpts	8
Data analysis	S14	Process by which inferences, themes, etc., were identified and developed, including the researchers involved in data analysis; usually references a specific paradigm or approach; rationale [b]	7-8
Techniques to enhance trustworthiness	S15	Techniques to enhance trustworthiness and credibility of data analysis (e.g., member checking, audit trail, triangulation); rationale	8
**RESULTS/FINDINGS**			
Synthesis and interpretation	S16	Main findings (e.g., interpretations, inferences, and themes); might include development of a theory or model, or integration with prior research or theory	9-15
Links to empirical data	S17	Evidence (e.g., quotes, field notes, text excerpts, photographs) to substantiate analytic findings	9-15
**DISCUSSION**			
Integration with prior work, implications, transferability, and contribution(s) to the field	S18	Short summary of main findings; explanation of how findings and conclusions connect to, support, elaborate on, or challenge conclusions of earlier scholarship; discussion of scope of application/ generalizability; identification of unique contribution(s) to scholarship in a discipline or field	15-17
Limitations	S19	Trustworthiness and limitations of findings	17
**OTHER**			
Conflicts of interest	S20	Potential sources of influence or perceived influence on study conduct and conclusions; how these were managed	18
Funding	S21	Sources of funding and other support; role of funders in data collection, interpretation, and reporting	18

[a] The authors created the SRQR by searching the literature to identify guidelines, reporting standards, and critical appraisal criteria for qualitative research; reviewing the reference lists of retrieved sources; and contacting experts to gain feedback. The SRQR aims to improve the transparency of all aspects of qualitative research by providing clear standards for reporting qualitative research.

[b] The rationale should briefly discuss the justification for choosing that theory, approach, method, or technique rather than other options available, the assumptions and limitations implicit in those choices, and how those choices influence study conclusions and transferability. As appropriate, the rationale for several items might be discussed together.

For peer review only

BMJ Open

“In fact, that’s when I stopped using contraception”: A qualitative study exploring women’s experiences of using contraceptive methods in KwaZulu-Natal, South Africa

Journal:	*BMJ Open*
Manuscript ID	bmjopen-2022-063034.R2
Article Type:	Original research
Date Submitted by the Author:	16-Feb-2023
Complete List of Authors:	Hlongwa, Mbuzeleni; University of KwaZulu-Natal College of Health Sciences, Nursing and Public Health<br>Mutambo, Chipo; University of KwaZulu-Natal College of Health Sciences<br>Hlongwana, Khumbulani; University of KwaZulu-Natal College of Health Sciences, Nursing and Public Health; University of KwaZulu-Natal College of Health Sciences, Public Health Medicine
**Primary Subject Heading**:	Reproductive medicine
Secondary Subject Heading:	Public health
Keywords:	Public health < INFECTIOUS DISEASES, Sexual and gender disorders < PSYCHIATRY, REPRODUCTIVE MEDICINE

SCHOLARONE™
Manuscripts

I, the Submitting Author has the right to grant and does grant on behalf of all authors of the Work (as defined in the below author licence), an exclusive licence and/or a non-exclusive licence for contributions from authors who are: i) UK Crown employees; ii) where BMJ has agreed a CC-BY licence shall apply, and/or iii) in accordance with the terms applicable for US Federal Government officers or employees acting as part of their official duties; on a worldwide, perpetual, irrevocable, royalty-free basis to BMJ Publishing Group Ltd ("BMJ") its licensees and where the relevant Journal is co-owned by BMJ to the co-owners of the Journal, to publish the Work in this journal and any other BMJ products and to exploit all rights, as set out in our licence.

The Submitting Author accepts and understands that any supply made under these terms is made by BMJ to the Submitting Author unless you are acting as an employee on behalf of your employer or a postgraduate student of an affiliated institution which is paying any applicable article publishing charge ("APC") for Open Access articles. Where the Submitting Author wishes to make the Work available on an Open Access basis (and intends to pay the relevant APC), the terms of reuse of such Open Access shall be governed by a Creative Commons licence – details of these licences and which Creative Commons licence will apply to this Work are set out in our licence referred to above.

Other than as permitted in any relevant BMJ Author's Self Archiving Policies, I confirm this Work has not been accepted for publication elsewhere, is not being considered for publication elsewhere and does not duplicate material already published. I confirm all authors consent to publication of this Work and authorise the granting of this licence.

For peer review only

"In fact, that's when I stopped using contraception": A qualitative study exploring women's experiences of using contraceptive methods in KwaZulu-Natal, South Africa

Mbuzeleni Hlongwa[1, 2], Chipo Mutambo[1], Khumbulani Hlongwana[1]

[1]Discipline of Public Health, School of Nursing and Public Health, University of KwaZulu-Natal, Durban, South Africa.

[2]Burden of Disease Research Unit, South African Medical Research Council, Cape Town, South Africa

Corresponding author:
Mbuzeleni Hlongwa
Discipline of Public Health Medicine,
School of Nursing and Public Health, University of KwaZulu-Natal, Howard College Campus, Glenwood, Durban, 4041, South Africa
hlongwa.mbu@gmail.com

For peer review only

For peer review only - http://bmjopen.bmj.com/site/about/guidelines.xhtml

Abstract

Objectives: This study explored women's experiences of using contraceptive methods in KwaZulu-Natal, South Africa.

Setting: In October 2021, we conducted a qualitative study at Umlazi Township in KwaZulu-Natal province, South Africa, through face-to-face in-depth interviews.

Participants: Fifteen women from four primary health care facilities were recruited through a criterion-based sampling strategy. Using NVivo version 11, two skilled researchers independently conducted thematic data analysis, as a mechanism for quality assurance, before the results were collated and reconciled.

Results: The study included 15 female participants, aged between 18 and 35 years, of whom two-thirds were aged 18-24 years. We found that women were concerned about unpleasant contraceptive side effects such as prolonged or irregular menstrual periods, bleeding, weight gain, and/or severe pains, resulting in discontinuation of their use. In addition to stockouts, women indicated that health care providers did not appropriately counsel or inform them about the available contraceptive methods, including how to use them.

Conclusions: Interventions aimed at reducing contraceptive stockouts are required to ensure that women are empowered to choose contraception based on their own preference, convenience, and/or experience. It is imperative that counselling on contraceptive methods' side effects be improved, to ensure that women have freedom to make informed decisions about their preferred method, proper management of side effects, and to assist them with method switching as needed, instead of discontinuation.

Keywords: Women, experiences, contraceptive use, discontinuation, KwaZulu-Natal, South Africa

For peer review only

Strengths and limitations of this study

- Use of in-depth qualitative interviews elicited rich data that offer important insights into sexually active women's discontinuation of contraceptive use.
- Given that this study focused on women attending public health care facilities, women who do not frequently use public health care clinics may have been excluded, hence their insights would be missing.
- The findings of this study were sought from participants' self-reported sexual and reproductive health information, rendering them prone to social desirability bias.
- Given the sensitivity of the topic, information prone to moral judgements, may have been withheld by the participants for image preservation.

For peer review only

For peer review only - http://bmjopen.bmj.com/site/about/guidelines.xhtml

Introduction

Contraception is one of the most important public health interventions which respond to sexual and reproductive health needs of women, thereby enabling them to plan their pregnancies and decide on the number of children they desire (1). The consistent and correct use of contraception has far-reaching benefits to both individuals and societies, including the reduction of pregnancy-related morbidity and mortality, termination of pregnancies (ToPs), improving educational opportunities, and empowerment of women (2, 3). Furthermore, contraceptive use positively affects the overall health of women of reproductive age, as it empowers them to autonomously make decisions regarding their own sexual and reproductive health (4, 5). Studies have shown that empowered and educated women are more likely to make informed decisions about the use of contraception (6, 7).

Despite the notable improvements in the uptake of contraceptives by women of reproductive age globally, the Sub-Saharan African (SSA) region continues to record a high proportion of women who experience unplanned pregnancies every year (8). In SSA, unmet contraception needs for women of reproductive age continue to be a public health concern, with a substantial number of these women being unable to access and use their preferred methods of contraception, which jeopardizes their desire to circumvent unplanned pregnancies (9, 10). We defined an unmet need for contraception as the proportion of women who are either (a) not pregnant, (b) not postpartum amenorrhoeic, are considered fecund, want to delay their next pregnancy, or (c) are postpartum amenorrhoeic and their most recent birth within the previous two years was unwanted or mistimed, but they are not using a contraceptive method (11, 12). The rate of unmet needs for contraceptive use is more than 20% among sexually active women in SSA (13, 14).

The 2016 Demographic and Health Survey report showed that 55% and 60% of married women and sexually active women, respectively, used contraceptives in South Africa (11). At least 15% of women who are in-union, have an unmet need for contraception to prevent unplanned pregnancy, and the figure is slightly higher for sexually active women (19%). However, the KwaZulu-Natal province accounts for 18% and 21% of the national figures of unmet need for contraception, respectively, among in-union and sexually active women (11). In South Africa, more than 100 000 terminations of pregnancy were reported in designated facilities in the

For peer review only - http://bmjopen.bmj.com/site/about/guidelines.xhtml

2016/2017 financial year, with the KwaZulu-Natal accounting for 15% of these (15). Despite ToP being legal in South Africa through the Choice of Termination of Pregnancy Act (CTOPA), which was made public in 1996, and amended in 2008 (16), many ToPs are conducted illegally, contributing to high maternal morbidity and mortality rates (3, 17, 18). The aim of the act is to reduce maternal mortality from illegal termination of pregnancies, and permits pregnant women to terminate their pregnancy legally (16).

High rates of unplanned pregnancy have also been reported among women diagnosed with HIV in South Africa (19, 20). Risky sexual behaviours are also common among young women in this country, as well as in KwaZulu-Natal, including early sexual debut, sex under the influence of alcohol or drugs, multiple sexual partners, and inconsistent condom use, all of which contribute to high levels of unplanned pregnancy and sexually transmitted diseases, including HIV (21-26).

Factors contributing to high rates of unplanned pregnancy in resource-limited settings, including South Africa, are well documented. Insufficient knowledge of contraception, gender inequality, intimate partner violence, poverty, and inconsistent and incorrect use of contraception, are some of the factors (8, 27-29). Low levels of contraceptive use have been associated with age, low education level and low socioeconomic status, limited knowledge and inaccessibility of contraceptive methods, and resource-limited rural residential settings (23, 29-34). Among adolescent girls, some of the barriers to contraceptive use include the lack of desire to avoid, delay, space or limit childbearing; the lack of confidence and ability to seek and/or negotiate contraceptive use; the lack of desire to use contraception; and poor access to contraceptive services and to quality (35). Health system challenges, including contraception stockouts, long waiting times, and negative attitudes displayed by some health care providers, have also been reported to deter women, especially young women, from accessing contraceptive services from health care facilities, thereby contributing to low or inconsistent uptake of contraceptives (33, 36-38). High rates (29%) of contraceptive users discontinue using contraception within a year of starting to use contraception, due to side effects (28%), the desire to become pregnant (19%) or the desire for a more effective method (11%) (11). Contraceptive stockouts remain high in South Africa, deterring women from accessing preferred contraceptive methods in local public health care facilities, thereby contributing to low or inconsistent uptake of contraceptives (11, 39, 40).

For peer review only - http://bmjopen.bmj.com/site/about/guidelines.xhtml

Despite these challenges, the South African government has demonstrated commitment to ensuring universal access to contraception by women of reproductive age. In South Africa, only 46% of births occurring within a five-year period preceding the SADHS 2016, were intended at the conception time, while 34% and 20%, respectively, were mistimed and unwanted (11). This study explored women's experiences of using contraceptive methods in KwaZulu-Natal, South Africa. The use of qualitative research methods deepened our understanding of this phenomenon.

Methods

Study setting

Umlazi, a township populated with more than half a million people, is located in the province of KwaZulu-Natal (41) and is part of the eThekwini Metropolitan Municipality, which has the largest number of people on lifelong antiretroviral therapy (ART) (N=450 238) in the province, and accounts for 16.7% of HIV prevalence (42). Umlazi has 10 public health care clinics and one public health hospital. Four public health care clinics, spread across various parts of Umlazi, participated in the study.

Study design

We explored women's experiences of using contraceptive methods, using an exploratory descriptive qualitative study design (43). We generated data in October 2021, through face-to-face in-depth interviews with women of reproductive age accessing health care services, from four facilities in Umlazi.

Sampling

We employed criterion-based sampling strategies, to identify potentially eligible women from four primary health care facilities. In order to be eligible for selection, women had to meet the following criteria: (a) be of reproductive age (18–49 years); (b) reside in Umlazi Township; (c) be sexually active (women who had sexual intercourse within three months preceding data collection); and (d) use contraception or would have used contraception within three months preceding data collection. Women who were outside the age brackets (18-49 years), pregnant, or sexually inactive, were excluded from the study. Interviews were conducted after services had been rendered by the facility, in a designated quite area within the facility.

For peer review only - http://bmjopen.bmj.com/site/about/guidelines.xhtml

Data collection tool

We developed an in-depth interview guide in the English language and then translated it into IsiZulu, which is the dominant language used in Umlazi. A female research assistant with a track record of conducting interviews and qualitative research, was recruited and provided with refresher training prior to collecting the data. The research assistant is fluent in both English and IsiZulu languages. Interviews were audio-recorded (with participants' permission). To minimize any inconsistencies during the data collection period, the interview guide was pre-tested with five participants who did not form part of the study. The scope of inquiry of the interviews focused on four key components of contraception, namely: (i) awareness about different contraceptive methods, (ii) access (availability/stockouts of preferred contraceptive methods, counselling), (iii) uptake (key considerations in deciding whether or not to start using contraception), and (iv) adherence/continuation (key considerations in deciding whether or not to continue or discontinue using contraception, including side effects, violence, stigma, discrimination, judgements).

Data collection

Data was collected iteratively to ensure that researchers engaged with preliminary data analysis of the information collected, learn emerging themes, identify gaps in the data, and adapt the data collection process for subsequent interviews. Each interview lasted for 30 to 60 minutes. Data saturation was reached at 15 interviews. At least five potential participants refused to participate in the study when they were approached. The lead author held regular debriefing meetings with the research assistant to discuss field experiences, challenges, emergent issues, lessons learned, and how she affected and/or was affected by the interviews. Data collection only took place after the services had been rendered by the facility. These participants were recruited regardless of the service they presented themselves for at the facility. We did not collect information regarding the care service they visited the facility for.

Data analysis

Using NVivo version 11, two researchers independently conducted the analysis from data coding to the development of themes, and this was done iteratively, guided by Richie and Spencer's framework (44). The framework outlines the following stages for conducting qualitative data analysis: (a) familiarisation with the data through reading all the transcripts and listening to the audio recordings; (b) generating initial codes using an open coding method,

For peer review only - http://bmjopen.bmj.com/site/about/guidelines.xhtml

where each segment of data relevant to this study's research objective was coded; (c) development of a thematic framework extracting key themes from the coded data; (d) application of the thematic framework to all the data; (e) charting of the data, enabling systematic comparisons between data sets; and (f) analysis of the charts for patterns and associations between and within each unit of analysis.

Data quality

To ensure rigor and accuracy, comparative data analysis was conducted by two skilled researchers, who independently read all the transcripts to gain an understanding of the content and scope of the data collected, prior to data coding. The outcome of coding was verified, cross-checked, and thoroughly discussed between the two members, to ensure that the research question was answered. We used the COREQ checklist to ensure that the study adhered to quality standards for reporting qualitative study findings (45).

Ethical considerations

Ethics approval and gatekeeper permission were obtained from the University of KwaZulu-Natal Biomedical Research Ethical Committee (BREC) (Ref No: BE424/18) and the Department of Health's National Health Research Database (NHRD) (Ref No: KZ_2018009_013), respectively. The eThekwini District's Ethical Review Committee also approved the study. The study was also supported by the participating health facilities. All participants who volunteered to participate in the study signed an informed consent form prior to their participation. The privacy and confidentiality of participants were protected.

Patient and Public Involvement

Patients or the public were not involved in the design, or conduct, or reporting, or dissemination plans of our research

Results

Background characteristics of study participants

The study included 15 women, aged between 18 and 35 years, with a median age of 23 years (Table 1). All participants were Black African women, with more than two-thirds (n=10) aged 18-24 years. The majority (n=12) had attained a secondary level of education and nearly half (n=7) were unemployed. Almost all participants (n=14) were not married.

For peer review only

For peer review only - http://bmjopen.bmj.com/site/about/guidelines.xhtml

Table 1: Demographic characteristics of participants, Umlazi Township, KwaZulu-Natal, South Africa, 2021

**Characteristic**	**Participants n=15**
**Age (years)**	18-49
Median (IQR)	23 (19-26)
**Age categories, n (%)**	
18-24 years	10 (67)
25-49 years	5 (33)
**Population group, n (%)**	
Black African	15 (100)
**Level of education, n (%)**	
Secondary	12 (80)
Tertiary	3 (20)
**Employment status, n (%)**	
Unemployed	7 (47)
Employed	4 (27)
Studying	4 (27)
**Marital status, n (%)**	
Not married	14 (93)
Married	1 (7)

Key themes

The following key themes were developed during analysis: negative effects of contraceptive methods; stockouts of preferred contraceptive methods: inconsistent or incorrect use of contraceptive methods; inadequate counselling on contraceptive methods; and misconceptions about contraception.

Negative effects of contraceptive methods

Some participants reported unpleasant side effects resulting using some contraceptive methods. The most common side effect reported by the participants, was prolonged menstrual periods resulting from using injectable contraceptives.

"The injection made me have prolonged periods than usual. I usual go on periods for three days, but after taking the injection my periods would last for seven days… I was tired of being on periods all the time. My periods lasted longer than usual. I'd keep having periods. Then it would stop eventually, but then again the following months when it's time for my periods, they would last longer… The next month, same thing". (19-year-old)

"I went on a prolonged menstrual cycle, my periods started from the 2nd to the 12th August… I don't know what is going on, I don't know…But the injection didn't treat me well. I had

For peer review only - http://bmjopen.bmj.com/site/about/guidelines.xhtml

started with the two months injection. But I stopped using the two months injection because it would make me go on periods at any time. It made me bleed at any time". (19-year-old)

Some women indicated that using the injectable contraceptive method made them bleed heavily, or that the injectable method interfered with their menstrual cycle and resulted in inconsistent menstrual periods.

She [nurse] had given me the 3 months injection. The three months injection was making me bleed a lot. My bleeding couldn't stop. Then I did some research and asked from others and they said that it happens that the injection would not respond well on your body". (20-year-old)

"It was not treating me well. It would make me go on periods at least twice in one month. I would go on periods for three or four days but twice a month. In the beginning it treated me well. Then it changed later on". (25-year-old)

Women reported that using contraception contributed to changes in their appetite for food, thereby affecting their weight. Some experienced weight gain while others lost weight.

"…when I started with the contraceptives, I gained a lot of weight and I was oily, like my face was messed up so I stopped and then after a year I went back [re-started contraception]…". (22-year-old)

"I started by using the three months injection, but it made me lose weight so bad, then I switched from it to the two months injection". (25-year-old)

Some participants reacted to side effects by simply discontinuing contraception, while others were forced to switch to a different contraceptive method.

"The injection made me have [more] extended periods than usual… So I didn't even ask, I just decided to stop using contraception. In fact, that's when I stopped using contraception. I stopped using contraception last year or last-of-last year". (19-year-old)

For peer review only - http://bmjopen.bmj.com/site/about/guidelines.xhtml

"Then I came back and told the nurse about the side effects I was experiencing while using that injection. I then asked her to switch me to a different method. And then she switched me to a two-months injection". (20-year-old)

Stockouts of preferred contraceptive methods

Participants reported stockouts of their preferred contraceptive methods as a pervasive challenge in the public health care facilities.

"Yes, even last week they said they were out of stock, especially the three months injection, they only had the two month's one". (18-year-old)

"…sometimes you come here and find that injection is not available… I don't know what can they do to improve the situation. I sometimes ask nurses why they don't have the injections and they say that it is the department that is not delivering them". (20-year-old)

Changing to an alternative contraceptive method was one of the options pursued by some participants whenever they were unable to receive their preferred contraceptive method, while others opted to wait for their preferred contraceptive method. Those who opted to wait for their contraceptive method of choice, had to modify their lifestyle during the period of being not protected by contraception.

"I continued using the three months injection. But it would happen that sometimes they would not have it at the clinic. Then I would be switched to the two months injection". (20-year-old)

"I decided to stop. I didn't take them. I stopped using contraceptives. My date was on the 12th September. I came here and I was told that the injection is not available. Then, again I came back on the 18th September. And they told me again that the injection is not available. Then I waited. Then I received a message from my friend who told me that she was at the clinic and that the injection is now available. I think it was last week. Then I came back and took the injection". (20-year-old)

"For me I don't want to use something else when I'm still going to use the implant. I don't want too many in my body. At least if I use one. I should not mix. I should just be patient for

For peer review only - http://bmjopen.bmj.com/site/about/guidelines.xhtml

the implant. I should be able to control myself. For now, I'm trying to avoid having sex, since August". (24-year-old)

The contraceptive methods stockouts was reported to have far-reaching implications on women, as it does not only make women vulnerable to unplanned pregnancies, but it also cause emotional discomfort. While others were discouraged to continue using contraception due to stockouts.

"During that time the injection is not available, you are not comfortable. You are scared. It is hard when the injection is not available". (20-year-old)

"You'll end up getting discouraged if you don't get the contraceptive methods you need. All they need to do is to ensure that the preferred contraceptive methods are always available". (35-year-old)

Inconsistent or incorrect use of contraceptive methods

Participants indicated that they sometimes could not consistently use contraceptives, as some of them missed their re-injection appointments. This resulted in some of them experiencing an unplanned pregnancy.

"…When my re-injection date had passed, as I was taking a break, so that my blood will flow and the side effects would stop, I then got pregnant. I had planned to wait for a month. Then I would start the following month. It was stupid I don't know how [laughs]. And then during that period I became pregnant. It just happened so quickly. I couldn't believe it. I couldn't believe that I was really pregnant". (23-year-old)

"I do have a child. I didn't stop using contraception, but I got the child after I skipped my date for re-injection. It just happened that I got pregnant". (25-year-old)

Most participants showed less preference for the contraceptive pills, fearing that they would not be able to maintain or use them consistently or correctly.

"…what made me stop using them [contraceptive pills] is because I used to miss them because you have to take them at the same time daily, I stopped taking them… I stopped using

For peer review only - http://bmjopen.bmj.com/site/about/guidelines.xhtml

them because I kept missing the time whereas you are supposed to take them at the same time". (19-year-old)

"I've never used something else. I've only used the injection. Because I cannot use the pills because I would forget them. I forget the pills I cannot use them". (23-year-old)

Some participants reported instances where health care providers either inserted the implant incorrectly, or inserted it into a pregnant woman, as they hardly ever checked a woman's pregnancy status before inserting the implant.

"…and I also got pregnant while the implant was inserted in me… Maybe they inserted it while I was already pregnant. They didn't check my pregnancy status. They didn't run pregnancy tests. They just inserted it". (19-year-old)

"I had pregnancy symptoms. I would vomit. I would experience things that I didn't understand. Then I went back to the clinic and I was told that the implant was not inserted correctly. Then they started telling me about the possible risks since I am already pregnant. That I might get a miscarriage. And I did get a miscarriage. I don't know if that's what caused the miscarriage. After they removed the implant, I had already lost hope because I was bleeding". (19-year-old)

Lack of counselling on contraceptive methods

Participants indicated that they were not counselled or given adequate information regarding the use of, and the possible side effects, of various contraceptive methods offered at health care facilities.

"…the nurse did not explain anything to me. She did not tell me how it works. I do have some questions about using contraception. I want to ask that since they are different, there's one for three months and another one for two months – what's the difference between them? That's all I want to ask". (18-year-old)

"She didn't even ask me which method I wanted to use or what. She didn't and I couldn't ask questions. I didn't even know which injection she was giving to me [after giving birth at the hospital]. She didn't say anything. She just calls your name and then gives you the injection….. It got me confused. Because I wanted to use a different method. But I only realized what they had given me when I was reading the card because it was written there. And there was nothing I could still do". (20-year-old)

For peer review only - http://bmjopen.bmj.com/site/about/guidelines.xhtml

"…there was no opportunity because in the room we were getting injections from, we were many so there was no space for discussing or asking questions. So you'd get the injection and go home. You didn't have time to discuss things or ask questions". (24-year-old)

Misconceptions about contraception

Concerns over the effectiveness of using contraceptive methods were raised, with some women reporting that they do not trust that the contraceptives are effective enough to prevent someone from getting an unplanned pregnancy.

"I think that because there's many different contraceptive methods, what I know about the injection is that it reduces chances of getting pregnant. But it is not 100% accurate…. I will continue using a condom because I know that the injection is not 100% sure." (18-year-old)

Some participants raised concerns about the possibility of contraceptive method impairing future fertility, with others preferring to wait until they got their first child before using contraceptive method.

"…but the very first time I heard about contraception, I was told by my mother who asked me to use contraception, so that I won't get pregnant. I hadn't had a child at that time. But I refused because I heard people saying that if you use contraception when you haven't had a child, it happens that you might not be able to conceive. So I didn't use contraception. I only started using contraception after getting a baby. I have one child". (20-year-old)

"I only used it for a short period because I was told that it's dangerous to use the injection when you don't have a child because it completely prevents you from getting pregnant in future….". (25-year-old)

Discussion

In this study, we adopted a qualitative approach to exploring sexually active women's experiences of using contraceptive methods in KwaZulu-Natal, South Africa . Our study participants shared crucial insights for understanding the challenges women face, when accessing and using contraceptive methods in Umlazi Township, KwaZulu-Natal. Our results show that negative effects of contraceptive methods; stockouts of preferred contraceptive

For peer review only - http://bmjopen.bmj.com/site/about/guidelines.xhtml

methods, inconsistent or incorrect use of contraceptive methods; inadequate counselling on contraceptive methods; and misconceptions about contraception, affect the use of contraceptives in the study setting, and this may have some relevance for other comparable settings.

Side effects play an important role in a woman's decision as to whether to continue or discontinue using contraception, as has been reported in other studies (46, 47). Similar to our study, prolonged or irregular menstrual cycle patterns have also been reported as one of the most common side effects that influence women to discontinue contraceptive use (47-49). Both the injectable users and implant users have concerns about bleeding side effects, which are known to have contributed to discontinuation of contraception in similar settings (47). Contraceptive discontinuation has dire implications, including unplanned pregnancy. Therefore, women should be empowered to avoid unplanned pregnancies, by identifying alternative contraceptive methods that meet their individual needs.

There have been concerns about the growing number of women who return to clinics for implant removal, only after a few months of insertion, due to changes in bleeding patterns or excessive bleeding (48, 50, 51). Side effects, notably prolonged or irregular bleeding, have also been reported by health care providers as the most common reason for early Implanon discontinuation (48). In addition to this, other factors have been reported to influence women to remove the implant, including incorrect positioning and low quality of care (52-54). Incorrect positioning of the implant was also reported in this study as an important factor for its discontinuation. The implant protects against pregnancy for up to three years before it needs to be replaced, and this reduces frequent clinic visits among users (55). However, the growing number of women removing the implant given the side effects, suggests the need for pre-insertion counselling, proper management, and empowerment of women through information regarding alternative contraceptive options for better decision-making (51).

Contraceptive stockouts occur when one or more contraceptive methods are not available at a health care facility that routinely provides that method. Contraceptive methods stockouts are one of the main factors contributing to contraceptive switching and discontinuation among women (56, 57). Contraceptive methods should be available at all levels of health care, but not

For peer review only - http://bmjopen.bmj.com/site/about/guidelines.xhtml

all health care facilities offer the full mix of modern contraceptive methods. Women have different contraceptive method preferences and experiences; therefore, a wide variety of contraceptive methods should always be available; to ensure that women are empowered to choose contraception based on their own preference, convenience, and/or experience. It has been reported that the use of modern contraception increases when more methods become available (58). Given the high rates of contraceptive method stockouts in South Africa, unemployed women are likely to suffer the most, due to financial constraints.

Health care providers play an important role in ensuring access to contraceptive methods among women. As such, health care providers have an important responsibility in educating women and providing complete information about possible side effects, and the effectiveness of their preferred method. It is imperative that counselling on side effects be improved, to ensure that women have freedom to make informed decisions about their preferred method, and to assist them with method switching as needed. However, counselling when receiving family planning services is limited in South Africa (40). Moreover, a single face-to-face counselling appointment in a busy facility may not be enough to convey all of the information a woman requires about: the reproductive cycle, returning to fertility after discontinuing a method, potential drug-drug interactions, the need for dual protection, and so on (40). In addition to traditional face-to-face interactions between health care providers and contraceptive users, innovative approaches, such as internet-based sources of information, text message reminders, and brochures are needed to improve women's comprehension of how contraceptive methods work (40, 59, 60).

Our study has important limitations to note. The findings of this study were sought from participants' self-reported sexual and reproductive health information, rendering them prone to social desirability bias. Given the sensitivity of the topic, information deemed to have the potential for leading to judgements, may have been withheld by the participants for image preservation. Given that this study focused on women attending public health care facilities, women who do not frequently use public health care clinics may have been excluded, hence their insights would be missing. Furthermore, the study planned to include participants aged 18-49 years. However, we strangely did not get women who were aged 36-49 years. In retrospect, this is something that we could have interrogated further. Lastly, due to the

For peer review only - http://bmjopen.bmj.com/site/about/guidelines.xhtml

qualitative nature of the study, the fact that this study was confined to limited health care facilities and participants, meant that our study findings cannot be generalised to other settings. However, the study provides important insights regarding the perceptions and experiences of contraceptive use among sexually active women in Umlazi Township, KwaZulu-Natal, South Africa.

Conclusion

Our findings illustrate that concerted efforts are urgently required to address women's concerns regarding the side effects arising from using contraceptive methods, as well as contraceptive stockouts, given the dire implications these may have on contraceptive discontinuation and subsequent unplanned pregnancy. The provision of comprehensive counselling services to support women who are having short-term side effects is paramount, to ensure that they can deal with side effects, or switch to a different method instead of completely discontinuing contraceptive use, to avoid unplanned pregnancy.

For peer review only

Declarations

Ethics approval and consent to participate: Ethics approval was obtained through the Biomedical Research Ethics Committee from the University of KwaZulu-Natal (Ref No: BE424/18). Approval was obtained through the National Health Research Database from the KwaZulu-Natal Provincial Department of Health (Ref No: KZ_2018009_013).

Consent for publication: Not applicable.

Availability of data and materials: All the data analysed and reported in this paper will be made available upon request.

Competing interest: The authors declare that they have no competing interests.

Funding: The work reported herein was made possible through funding by the South African Medical Research Council through its Division of Research Capacity Development under the **BONGANI MAYOSI NATIONAL HEALTH SCHOLARS PROGRAM** from funding received from the South African National Treasury. The content hereof is the sole responsibility of the authors and does not necessarily represent the official views of the SAMRC or the funders.

Authors' contributions: MH conceptualized and designed the study, as well as prepared the initial draft. KH and CM reviewed the manuscript. All the authors approved the final version of the manuscript.

Acknowledgements: The authors would like to thank the School of Nursing and Public Health, University of KwaZulu-Natal, Durban, South Africa and the Division of Research Capacity Development at SA MRC.

For peer review only - http://bmjopen.bmj.com/site/about/guidelines.xhtml

References

1. Bongaarts J, Cleland JC, Townsend J, Bertrand JT, Gupta MD. Family planning programs for the 21st century: rationale and design. 2012.
2. Michalow J, Chola L, McGee S, Tugendhaft A, Pattinson R, Kerber K, et al. Triple return on investment: the cost and impact of 13 interventions that could prevent stillbirths and save the lives of mothers and babies in South Africa. BMC Pregnancy and Childbirth 2015;15(1):1-13.
3. Darroch JE, Woog V, Bankole A, Ashford LS. Adding it up: costs and benefits of meeting the contraceptive needs of adolescents. 2016.
4. Ganatra B, Faundes A. Role of birth spacing, family planning services, safe abortion services and post-abortion care in reducing maternal mortality. Best Practice & Research Clinical Obstetrics & Gynaecology. 2016;36:145-55.
5. Hameed W, Azmat SK, Ali M, Sheikh MI, Abbas G, Temmerman M, et al. Women's empowerment and contraceptive use: the role of independent versus couples' decision-making, from a lower middle income country perspective. PloS one. 2014;9(8):e104633.
6. Corroon M, Speizer IS, Fotso J-C, Akiode A, Saad A, Calhoun L, et al. The role of gender empowerment on reproductive health outcomes in urban Nigeria. Maternal and child health journal. 2014;18(1):307-15.
7. Tekelab T, Melka AS, Wirtu D. Predictors of modern contraceptive methods use among married women of reproductive age groups in Western Ethiopia: a community based cross-sectional study. BMC women's health. 2015;15(1):1-8.
8. Cleland J, Harbison S, Shah IH. Unmet need for contraception: issues and challenges. Studies in family planning. 2014;45(2):105-22.
9. United Nations. Family Planning and the 2030 Agenda for Sustainable Development. New York: United Nations, Department of Economic and Social Affairs, Population Division.; 2019.
10. Chersich MF, Wabiri N, Risher K, Shisana O, Celentano D, Rehle T et al. Contraception coverage and methods used among women in South Africa: A national household survey. South African Medical Journal 2017;107(4):307-14.
11. NDoH, Stats SA, SAMRC, ICF. South Africa Demographic and Health Survey 2016. Pretoria, South Africa, and Rockville, Maryland, USA: National Department of Health (NDoH), Statistics South Africa (Stats SA), South African Medical Research Council (SAMRC), ICF; 2019.
12. Sedgh G, Ashford LS, Hussain R. Unmet need for contraception in developing countries: examining women’s reasons for not using a method. 2016.
13. Darroch JE, Sully E, Biddlecom A. Adding it up: investing in contraception and maternal and newborn health, 2017—supplementary tables. New York, NY: The Guttmacher Institute. 2017.
14. World Health Organization. Contraception. Geneva, Switzerland: World Health Organization; 2019.
15. SA National Department of Health. Pregancy terminations in designated South African facilities. Pretoria: SA National Department of Health; 2017.
16. Republic of South Africa. Choice on Termination of Pregnancy Act (No. 92 of 1996). Government Gazette. 1996;377(17602).
17. Hodes R. The culture of illegal abortion in South Africa. Journal of Southern African Studies. 2016;42(1):79-93.
18. Seidu A-A, Ahinkorah BO, Agbemavi W, Amu H, Bonsu F. Reproductive health decision-making capacity and pregnancy termination among Ghanaian women: Analysis of the 2014 Ghana demographic and health survey. Journal of Public Health. 2021;29(1):85-94.
19. Adeniyi OV, Ajayi AI, Moyaki MG, Ter Goon D, Avramovic G, Lambert J. High rate of unplanned pregnancy in the context of integrated family planning and HIV care services in South Africa. BMC health services research. 2018;18(1):1-8.

For peer review only

For peer review only - http://bmjopen.bmj.com/site/about/guidelines.xhtml

20. Schwartz SR, Rees H, Mehta S, Venter WDF, Taha TE, Black V. High incidence of unplanned pregnancy after antiretroviral therapy initiation: findings from a prospective cohort study in South Africa. PloS one. 2012;7(4):e36039.
21. Hlongwa M, Peltzer K, Hlongwana K. Risky sexual behaviours among women of reproductive age in a high HIV burdened township in KwaZulu-Natal, South Africa. BMC Infectious Diseases. 2020;20(1):1-9.
22. Toska E, Cluver LD, Boyes M, Pantelic M, Kuo C. From 'sugar daddies' to 'sugar babies': exploring a pathway among age-disparate sexual relationships, condom use and adolescent pregnancy in South Africa. Sexual health. 2015;12(1):59-66.
23. Christofides NJ, Jewkes RK, Dunkle KL, Nduna M, Shai NJ, Sterk C. Early adolescent pregnancy increases risk of incident HIV infection in the Eastern Cape, South Africa: a longitudinal study. Journal of the International AIDS Society. 2014;17(1):18585.
24. Atuyambe LM, Kibira SP, Bukenya J, Muhumuza C, Apolot RR, Mulogo E. Understanding sexual and reproductive health needs of adolescents: evidence from a formative evaluation in Wakiso district, Uganda. Reproductive health. 2015;12(1):1-10.
25. Solanke BL. Factors influencing contraceptive use and non-use among women of advanced reproductive age in Nigeria. Journal of Health, Population and Nutrition. 2017;36(1):1-14.
26. Blackstone SR, Iwelunmor J. Determinants of contraceptive use among Nigerian couples: evidence from the 2013 Demographic and Health Survey. Contraception and reproductive medicine. 2017;2(1):1-8.
27. Maharaj P. Stalling contraception? Perspectives and experiences of sexually active women and men. Agenda. 2012;26(2):100-10.
28. Jewkes R, Morrell R, Christofides N. Empowering teenagers to prevent pregnancy: lessons from South Africa. Culture, health & sexuality. 2009;11(7):675-88.
29. Chersich MF, Wabiri N, Risher K, Shisana O, Celentano D, Rehle T, et al. Contraception coverage and methods used among women in South Africa: A national household survey. South African Medical Journal 2017;107(4):307-14
30. Makola L, Mlangeni L, Mabaso M, Chibi B, Sokhela Z, Silimfe Z, et al. Predictors of contraceptive use among adolescent girls and young women (AGYW) aged 15 to 24 years in South Africa: results from the 2012 national population-based household survey. BMC women's health. 2019;19(1):1-7.
31. Digitale J, Psaki S, Soler-Hampejsek E, Mensch BS. Correlates of contraceptive use and health facility choice among young women in Malawi. The ANNALS of the American Academy of Political and Social Science. 2017;669(1):93-124.
32. Nyarko SH. Prevalence and correlates of contraceptive use among female adolescents in Ghana. BMC women's health. 2015;15(1):1-6.
33. Hlongwa M, Mashamba-Thompson T, Makhunga S, Hlongwana K. Evidence on factors influencing contraceptive use and sexual behavior among women in South Africa: A scoping review. Medicine. 2020;99(12):e19490.
34. Hlongwa M, Kalinda C, Peltzer K, Hlongwana K. Factors associated with modern contraceptive use: a comparative analysis between younger and older women in Umlazi Township, KwaZulu-Natal, South Africa. Women's Health. 2021;17:17455065211060641.
35. Chandra-Mouli V, Akwara E. Improving access to and use of contraception by adolescents: what progress has been made, what lessons have been learnt, and what are the implications for action? Best Practice & Research Clinical Obstetrics & Gynaecology. 2020;66:107-18.
36. Hlongwa M, Tlou B, Hlongwana K. Healthcare providers' knowledge and perceptions regarding the use of modern contraceptives among adolescent girls in Umlazi Township, KwaZulu-Natal province, South Africa. The Pan African Medical Journal. 2021;38.
37. Smith P, Marcus R, Bennie T, Nkala B, Nchabeleng M, Latka M, et al. What do South African adolescents want in a sexual health service? Evidence from the South African Studies on HIV in Adolescents (SASHA) project. South African Medical Journal. 2018;108(8).

For peer review only - http://bmjopen.bmj.com/site/about/guidelines.xhtml

38. Jonas K, Duby Z, Maruping K, Dietrich J, Slingers N, Harries J, et al. Perceptions of contraception services among recipients of a combination HIV-prevention interventions for adolescent girls and young women in South Africa: a qualitative study. Reproductive Health. 2020;17(1):1-14.
39. Blanc AK, Curtis SL, Croft TN. Monitoring contraceptive continuation: links to fertility outcomes and quality of care. Studies in family planning. 2002;33(2):127-40.
40. Lince-Deroche N, Pleaner M, Morroni C, Mullick S, Firnhaber C, Harries J, et al. Achieving universal access to sexual and reproductive health services: the potential and pitfalls for contraceptive services in South Africa. South African Health Review. 2016;2016(1):95-108.
41. Statistics South Africa. Census 2011. Pretoria: Statistics South Africa; 2012.
42. Simbayi LC, Zuma K, Zungu N, Moyo S, Marinda E, Jooste S, et al. South African National HIV Prevalence, Incidence, Behaviour and Communication Survey, 2017. Cape Town: HSRC; 2019.
43. Parker I. Discourse analysis: Dimensions of critique in psychology. Qualitative Research in Psychology. 2013;10(3):223-39.
44. Ritchie J, Spencer L. Qualitative data analysis for applied policy research. Analyzing qualitative data: Routledge; 2002. p. 187-208.
45. Tong A, Sainsbury P, Craig J. Consolidated criteria for reporting qualitative research (COREQ): a 32-item checklist for interviews and focus groups. International journal for quality in health care. 2007;19(6):349-57.
46. Castle S, Askew I. Contraceptive discontinuation: Reasons, challenges and solutions: Population Council. FP2020. 2015.
47. Keogh SC, Otupiri E, Castillo PW, Chiu DW, Polis CB, Nakua EK, et al. Hormonal contraceptive use in Ghana: the role of method attributes and side effects in method choice and continuation. Contraception. 2021.
48. Adeagbo O, Mullick S, Pillay D, Chersich M, Morroni C, Naidoo N, et al. Uptake and early removals of Implanon NXT in South Africa: Perceptions and attitudes of healthcare workers. South African Medical Journal. 2017;107(10).
49. Veisi F, Zangeneh M. Comparison of two different injectable contraceptive methods: Depo-medroxy progesterone acetate (DMPA) and cyclofem. Journal of family & reproductive health. 2013;7(3):109.
50. Burusie A. Reasons for premature removal of Implanon among users in Arsi zone, Oromia region, Ethiopia, 2013. Reproductive System & Sexual Disorders: Current Research. 2015;4(1):1-6.
51. Mrwebi KP, Ter Goon D, Owolabi EO, Adeniyi OV, Seekoe E, Ajayi AI. Reasons for discontinuation of Implanon among users in Buffalo City Metropolitan Municipality, South Africa: a cross-sectional study. African journal of reproductive health. 2018;22(1):113-9.
52. Zerihun H, Biru A, Worku A, Desta E, Tafesse H, Dinssa H, et al. Implanon removal experiences of women in Butajira, Ethiopia. The Ethiopian Journal of Health Development. 2015;29(3).
53. Birhane K, Hagos S, Fantahun M. Early discontinuation of implanon and its associated factors among women who ever used implanon in Ofla District, Tigray, Northern Ethiopia. International Journal of Pharma Sciences and Research. 2015;6(3):544-51.
54. Graesslin O, Korver T. The contraceptive efficacy of Implanon®: A review of clinical trials and marketing experience. The European Journal of Contraception & Reproductive Health Care. 2008;13(sup1):4-12.
55. Bhatia P, Nangia S, Aggarwal S, Tewari C. Implanon: subdermal single rod contraceptive implant. The Journal of Obstetrics and Gynecology of India. 2011;61(4):422.
56. Burke H, Ambasa-Shisanya C. Qualitative study of reasons for discontinuation of injectable contraceptives among users and salient reference groups in Kenya. African journal of reproductive health. 2011;15(2).

For peer review only - http://bmjopen.bmj.com/site/about/guidelines.xhtml

57. Daff BM, Seck C, Belkhayat H, Sutton P. Informed push distribution of contraceptives in Senegal reduces stockouts and improves quality of family planning services. Global Health: Science and Practice. 2014;2(2):245-52.

58. Ross J, Stover J. Use of modern contraception increases when more methods become available: analysis of evidence from 1982–2009. Global Health: Science and Practice. 2013;1(2):203-12.

59. Trent M, Thompson C, Tomaszewski K. Text messaging support for urban adolescents and young adults using injectable contraception: outcomes of the DepoText pilot trial. Journal of Adolescent Health. 2015;57(1):100-6.

60. Lou C-H, Wang B, Shen Y, Gao E-S. Effects of a community-based sex education and reproductive health service program on contraceptive use of unmarried youths in Shanghai. Journal of Adolescent Health. 2004;34(5):433-40.

For peer review only

Standards for Reporting Qualitative Research: A Synthesis of Recommendations

TOPIC	NO.	ITEM	REPORTED ON PAGE #
**TITLE**			
Title	S1	Concise description of the nature and topic of the study Identifying the study as qualitative or indicating the approach (e.g., ethnography, grounded theory) or data collection methods (e.g., interview, focus group) is recommended	1
Abstract	S2	Summary of key elements of the study using the abstract format of the intended publication; typically includes background, purpose, methods, results, and conclusions	2
**INTRODUCTION**			
Problem formulation	S3	Description and significance of the problem/phenomenon studied; review of relevant theory and empirical work; problem statement	4-5
Purpose or research question	S4	Purpose of the study and specific objectives or questions	6
**METHODS**			
Qualitative approach and research paradigm	S5	Qualitative approach (e.g., ethnography, grounded theory, case study, phenomenology, narrative research) and guiding theory if appropriate; identifying the research paradigm (e.g., postpositivist, constructivist/ interpretivist) is also recommended; rationale [b]	6
Researcher characteristics and reflexivity	S6	Researchers' characteristics that may influence the research, including personal attributes, qualifications/experience, relationship with participants, assumptions, and/or presuppositions; potential or actual interaction between researchers' characteristics and the research questions, approach, methods, results, and/or transferability	6-7
Context	S7	Setting/site and salient contextual factors; rationale [b]	6
Sampling strategy	S8	How and why research participants, documents, or events were selected; criteria for deciding when no further sampling was necessary (e.g., sampling saturation); rationale [b]	6
Ethical issues pertaining to human subjects	S9	Documentation of approval by an appropriate ethics review board and participant consent, or explanation for lack thereof; other confidentiality and data security issues	8
Data collection methods	S10	Types of data collected; details of data collection procedures including (as appropriate) start and stop dates of data collection and analysis, iterative process, triangulation of sources/methods, and modification of procedures in response to evolving study findings; rationale [b]	7
Data collection instruments and technologies	S11	Description of instruments (e.g., interview guides, questionnaires) and devices (e.g., audio recorders) used for data collection; if/how the instrument(s) changed over the course of the study	7

For peer review only

TOPIC	NO.	ITEM	REPORTED ON PAGE #
Units of study	S12	Number and relevant characteristics of participants, documents, or events included in the study; level of participation (could be reported in results)	9
Data processing	S13	Methods for processing data prior to and during analysis, including transcription, data entry, data management and security, verification of data integrity, data coding, and anonymization/deidentification of excerpts	8
Data analysis	S14	Process by which inferences, themes, etc., were identified and developed, including the researchers involved in data analysis; usually references a specific paradigm or approach; rationale [b]	7-8
Techniques to enhance trustworthiness	S15	Techniques to enhance trustworthiness and credibility of data analysis (e.g., member checking, audit trail, triangulation); rationale	8
**RESULTS/FINDINGS**			
Synthesis and interpretation	S16	Main findings (e.g., interpretations, inferences, and themes); might include development of a theory or model, or integration with prior research or theory	9-15
Links to empirical data	S17	Evidence (e.g., quotes, field notes, text excerpts, photographs) to substantiate analytic findings	9-15
**DISCUSSION**			
Integration with prior work, implications, transferability, and contribution(s) to the field	S18	Short summary of main findings; explanation of how findings and conclusions connect to, support, elaborate on, or challenge conclusions of earlier scholarship; discussion of scope of application/ generalizability; identification of unique contribution(s) to scholarship in a discipline or field	15-17
Limitations	S19	Trustworthiness and limitations of findings	17
**OTHER**			
Conflicts of interest	S20	Potential sources of influence or perceived influence on study conduct and conclusions; how these were managed	18
Funding	S21	Sources of funding and other support; role of funders in data collection, interpretation, and reporting	18

[a] The authors created the SRQR by searching the literature to identify guidelines, reporting standards, and critical appraisal criteria for qualitative research; reviewing the reference lists of retrieved sources; and contacting experts to gain feedback. The SRQR aims to improve the transparency of all aspects of qualitative research by providing clear standards for reporting qualitative research.

[b] The rationale should briefly discuss the justification for choosing that theory, approach, method, or technique rather than other options available, the assumptions and limitations implicit in those choices, and how those choices influence study conclusions and transferability. As appropriate, the rationale for several items might be discussed together.

For peer review only
